# Production, purification, and radiolabeling of the ^203^Pb/^212^Pb theranostic pair

**DOI:** 10.1186/s41181-021-00121-4

**Published:** 2021-02-01

**Authors:** Brooke L. McNeil, Andrew K. H. Robertson, Winnie Fu, Hua Yang, Cornelia Hoehr, Caterina F. Ramogida, Paul Schaffer

**Affiliations:** 1 Life Sciences Division, TRIUMF, Vancouver, BC Canada; 2 Department of Chemistry, Simon Fraser University, Burnaby, BC Canada; 3 Department of Physics and Astronomy, University of British Columbia, Vancouver, BC Canada; 4 Department of Radiology, University of British Columbia, Vancouver, BC Canada

**Keywords:** Lead-212, Lead-203, Thorium-228 generator, Thallium-203, Theranostic, Cyclen, DOTA, Pyridyl, Chelators, Radiolabeling

## Abstract

**Background:**

Lead-212 (^212^Pb, t_1/2_ = 10.6 h) and lead-203 (^203^Pb, t_1/2_ = 51.9 h) are an element-equivalent, or a matched theranostic radioisotope pair that show great potential for application in targeted radionuclide therapy (TRT) and single-photon emission computed tomography (SPECT), respectively. At TRIUMF we have produced both ^203^Pb and ^212^Pb using TRIUMF’s TR13 (13 MeV) and 500 MeV cyclotrons, and subsequently purified and evaluated both radioisotopes using a series of pyridine-modified DOTA analogues in comparison to the commercially available chelates DOTA (1,4,7,10-tetraazacyclododecane-1,4,7,10-tetraacetic acid) and TCMC (1,4,7,10-tetraaza-1,4,7,10-tetra(2-carbamoylmethyl)cyclododecane).

**Results:**

Proton irradiation (12.8 MeV) of natural and enriched thallium-203 (^203^Tl) targets gave ^203^Pb saturation yields of 134 ± 25 and 483 ± 3 MBq/μA, respectively. Thorium-228 (^228^Th, t_1/2_ = 1.9 y), a by-product of ^232^Th proton spallation on TRIUMF’s main 500 MeV beamline (beamline 1A, BL1A), was recovered to build a ^228^Th/^212^Pb generator with the ability to deliver up to 9–10 MBq of ^212^Pb daily. Both lead isotopes were purified via solid phase extraction chromatography (Pb resin), and isolated in an acetate form ([^203/212^Pb]Pb(OAc)_2_) suitable for direct radiolabeling of chelators and bioconjugates. A series of cyclen-based chelators (herein referred to as DOTA-1Py, -2Py, and -3Py) along with established chelates DOTA and TCMC were evaluated for their ability to complex both ^203^Pb and ^212^Pb. All chelates incorporated ^212^Pb/^203^Pb efficiently, with higher radiolabeling yields observed for the ^212^Pb-complexes.

**Conclusion:**

The production of ^203^Pb and ^212^Pb was established using TRIUMF 13 MeV and 500 MeV cyclotrons, respectively. Both production methods provided radiometals suitable for subsequent radiolabeling reactions using known and novel chelates. Furthermore, the novel chelate DOTA-3Py may be a good candidate for biomolecule conjugation and further theranostic ^212^Pb/^203^Pb studies.

**Supplementary Information:**

The online version contains supplementary material available at 10.1186/s41181-021-00121-4.

## Background

The fields of molecular imaging (MI) and targeted radionuclide therapy (TRT) rely on incorporating radioisotopes onto biomolecules that show high affinity for cancer cells in order to impart diagnostic and/or therapeutic information to health researchers and clinicians. Advances in understanding the molecular processes that define normal and aberrant cell behavior has led to the identification of an increasing number of biomolecular targets that can be exploited for targeted delivery of imaging and therapeutic agents specific to diseased cells. With targeted compound delivery, one can minimize ambiguous diagnostic outcomes and/or undesirable side effects during treatment by avoiding uptake or damage induced by off-target radiopharmaceutical accumulation (Kumar Bharti et al. 2012; Bono et al. 2003). MI relies on radionuclides which emit photons, either directly (such as in electron capture [EC] decay) or indirectly (such as in positron [β^+^] decay), while nuclides that emit cytocidal particles (such as beta [β^−^], alpha [α] particles, or Meitner-Auger electrons) can be used for TRT. *Theranostic* radiopharmaceuticals represent a combination of both MI and TRT isotopes onto a common biomolecule that can be used to both image and then treat disease, leading to a potent compound pairing that allows for visualization of the molecular processes underpinning disease and verifies cellular target presence for subsequent therapy (Yordanova et al. 2017; Rösch et al. 2017).

In general, a theranostic pair of radionuclides comprise of two chemically similar isotopes, one which can be used for imaging, and the other for therapy (Elgqvist et al. 2014). When the theranostic pair is composed of radionuclides of two different elements, the biodistribution of the radiopharmaceutical may differ and thus any quantitative dosimetric information predicted from the diagnostic imaging results may not be reflective of the therapeutic agent; this discrepancy can be minimized with matched theranostic pairs (Elgqvist et al. 2014). Matched theranostic pairs utilize different isotopes of the same element for diagnosis and therapy, giving rise to identical chemical species and thus biodistribution, which can give further insight on the suitability of the radiopharmaceutical for a patient being assessed or treated (Yordanova et al. 2017). Only the different half lives and their effect on biodistribution may need to be considered.

Lead-203 (^203^Pb, t_1/2_ = 51.9 h) and lead-212 (^212^Pb, t_1/2_ = 10.6 h) are an element-equivalent matched theranostic pair that have generated significant interest for use in theranostic radiopharmaceutical development (Máthé et al. 2016). ^212^Pb emits two β^−^ particles and one α particle during its decay chain and can be used for therapy. ^203^Pb decays by electron capture to ground state thallium-203 (^203^Tl), followed by the emission of a gamma-photon (279 keV; 81%) that is compatible for single photon emission computed tomography (SPECT) imaging while the lack of radioactive daughter products simplifies dosimetry calculations (Horlock et al. 1975).

**Fig. 1:**
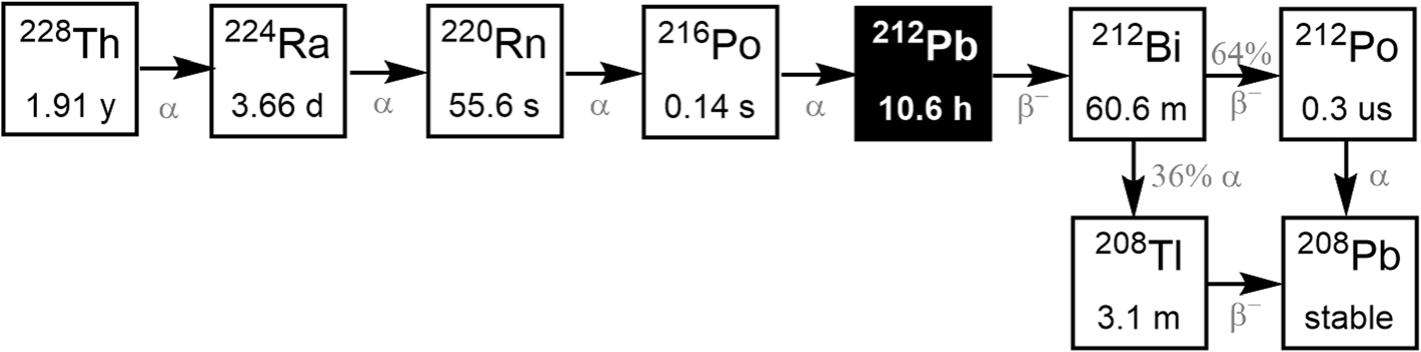
Decay scheme of ^228^Th to ^212^Pb and stable ^208^Pb

^212^Pb is a member of the uranium-232 (^232^U) and thorium-232 (^232^Th) decay chain, and is commonly produced by the decay of ^228^Th (t_1/2_ = 1.9 y) (Hassfjell 2001; Hassfjell and Hoff 1994; Zucchini and Friedman 1982) and radium-224 (^224^Ra, t_1/2_ = 3.64 days) (Mirzadeh 1998; Westrøm et al. 2017; Atcher et al. 1988; Bartoś et al. 2013; Milenic et al. 2015; Baidoo et al. 2013; Li et al. 2017) (Fig. 1). Many ^228^Th generators exploit the chemical or physical separation of the daughters ^224^Ra (Zucchini and Friedman 1982) and radon-220 (^220^Rn, t_1/2_ = 55.6 s) (Hassfjell 2001; Hassfjell and Hoff 1994) by using cation exchange columns (Zucchini and Friedman 1982) or chamber walls (Hassfjell and Hoff 1994), and glass bubblers (Hassfjell 2001), respectively. ^212^Pb has been collected using nitric (Hassfjell 2001) or hydrochloric (Zucchini and Friedman 1982) acid or water (Hassfjell and Hoff 1994) to give yields of 85–90%. However, many of the ^228^Th generators reported to date have difficulty providing practical quantities of ^212^Pb due to the radiolytic damage to the generator matrix material when higher levels of activity are included. To circumvent this, ^224^Ra generators have been used to produce ^212^Pb by separating ^224^Ra from ^228^Th on an anion exchange resin (Bartoś et al. 2013), followed by loading onto a cation (Mirzadeh 1998; Atcher et al. 1988; Bartoś et al. 2013; Milenic et al. 2015; Baidoo et al. 2013) exchange resin, actinide resin (Westrøm et al. 2017), or Pb-selective extraction resin (Li et al. 2017), from which ^212^Pb is eluted using HCl (Mirzadeh 1998; Westrøm et al. 2017; Atcher et al. 1988; Bartoś et al. 2013; Milenic et al. 2015; Baidoo et al. 2013) or a complexing agent (Li et al. 2017).

^203^Pb is a cyclotron produced isotope and can be prepared from charged particle (proton, deuteron, and alpha particle) bombardment of thallium (natural abundance 29.5% ^203^Tl, 70.5% ^205^Tl) (Horlock et al. 1975). At the end of bombardment, the thallium targets are dissolved in nitric acid (Horlock et al. 1975; Li et al. 2017; Henriksen and Hoff 1998; Garmestani et al. 2005) or a mix of nitric and hydrochloric acid (Máthé et al. 2016), before loading onto a Pb-selective extraction resin (Li et al. 2017; Henriksen and Hoff 1998; Garmestani et al. 2005), or anion exchange resin (Máthé et al. 2016). Alternatively, ^203^Pb is co-precipitated with Fe (OH)_3_ (Horlock et al. 1975) and eluted using complexing agents (Henriksen and Hoff 1998), dilute nitric acid (Li et al. 2017; Garmestani et al. 2005), or hydrochloric acid (Máthé et al. 2016). Many of the purification methods for both ^212^Pb and ^203^Pb produce large eluant volumes, of which the composition may be incompatible with radiolabeling, thus requiring evaporation and redissolution steps which can result in further reduction of yield or introduction of interfering stable impurities.

**Fig. 2:**
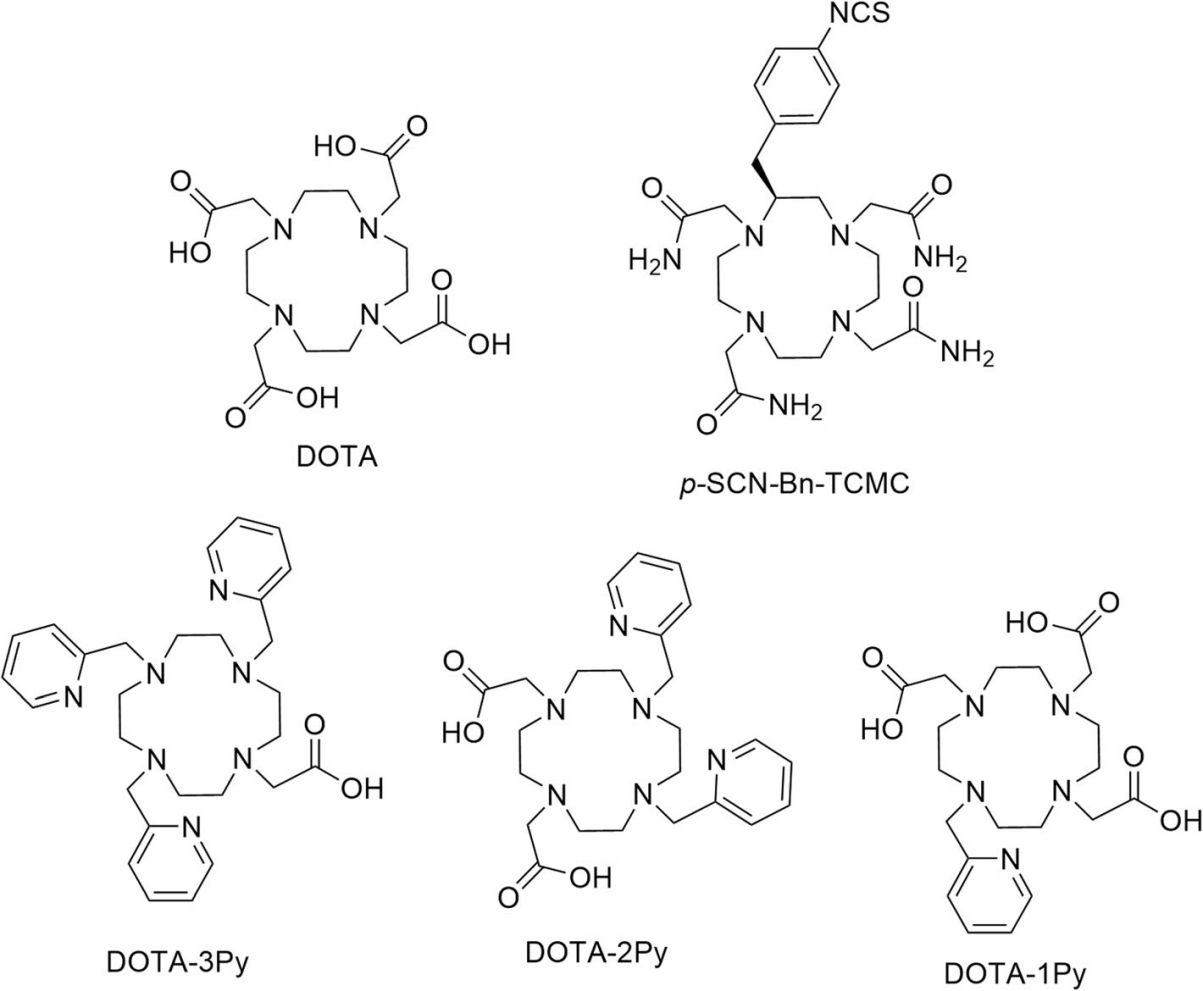
Chemical structures of commercially available Pb-chelators DOTA, *p-*SCN-Bn-TCMC, and pyridine-based cyclen analogues DOTA-1Py, DOTA-2Py, DOTA-3Py radiolabeled herein

In this study, extraction chromatography and small volumes of radiolabeling-compatible complexing agents are used to elute cyclotron produced ^203^Pb and ^228^Th-generator produced ^212^Pb to shorten processing time and allow direct radiolabeling of the purified ^203/212^Pb isotopes. A novel ^228^Th/^212^Pb generator concept was applied using a ^228^Th generator stock solution obtained via ^232^Th spallation on TRIUMF’s 500 MeV cyclotron. The preparation of the ^228^Th stock allowed direct loading onto Pb-selective resin, which returned ^228^Th directly to a storage vessel followed by elution of absorbed ^212^Pb with ammonium acetate (NH_4_OAc). We present the production of the ^203^Pb and ^212^Pb theranostic pair using TRIUMF’s TR13 (13 MeV) and 500 MeV cyclotron, subsequent radiochemical purification and isotope characterization. The separated radioisotopes were evaluated alongside each other for radiolabeling a series of TRIUMF developed, pyridine based-DOTA analogues (Yang et al. 2021) along with commercial standards DOTA and TCMC (Fig. 2), and the stability of each radiometal-complex was evaluated in vitro.

## Methods

### Materials and methods

All solvents and reagents were purchased from commercial suppliers (Sigma Aldrich, Fisher Scientific, VWR) and used as received, unless otherwise noted. Ultrapure nitric acid (Environmental Grade) was purchased from VWR and Ultrapure HCl (TraceSELECT), NaOH (99.99% trace metal grade), and ammonium acetate (ACS grade) were purchased from Fisher Scientific. Pb resin (Di-t-butylcyclohexano 18-crown-6, 100–150 μm particle size) was purchased from Eichrom Technologies (Lisle, IL). 1 mL polypropylene cartridges and 1/8″ polyethylene frits were purchased from United Chemical Technologies (Lewistown, PA). Natural Tl (99.99% metals basis) was purchased from Alfa Aesar (Tewksbury, MA) and enriched Tl (98.57 ± 0.05% ^203^Tl, 1.43 ± 0.05% ^205^Tl) was purchased from BWX Technologies (Vancouver, BC). Human serum and Dowex 1 × 8 chloride form anion exchange resin (100–200 mesh) was purchased from Sigma Aldrich (St. Louis, MO). DOTA and *p-*SCN-Bn-TCMC (referred to herein as TCMC) were purchased from Macrocyclics (Plano, TX). DOTA-1Py (2,2′,2″-(10-(pyridin-2-ylmethyl)-1,4,7,10-tetraazacyclododecane-1,4,7-triyl) triacetic acid), DOTA-2Py, (2,2′-(7,10-bis (pyridin-2-ylmethyl)-1,4,7,10-tetraazacyclododecane-1,4-diyl) diacetic acid) and DOTA-3Py (2-(4,7,10-tris (pyridin-2-ylmethyl)-1,4,7,10-tetraazacyclododecan-1-yl) acetic acid) were synthesized as previously described (Yang et al. 2021). Instant thin layer chromatography paper impregnated with silicic acid (iTLC-SA) was purchased from Agilent Technologies (Santa Clara, CA). Deionized water was prepared on site using a Millipore Direct-Q® 3UV water purification system. Nuclear magnetic resonance (NMR) spectra were obtained using MeOD, DMSO-d_6_, or D_2_O. Signals were measured relative to the signal of the solvent. NMR spectra were obtained using a Bruker 400 (400 MHz), Bruker 500 (500 MHz), or a Bruker 600 (600 MHz). Mass spectrometry was performed on an Agilent 6210 time-of-flight LC-MS spectrometer or an Advion expression LC-MS equipped with an electrospray source. All radioactivity measurements for the ^203^Pb purification from Tl targets and the ^228^Th/^212^Pb generator were performed using gamma ray spectroscopy on an N-type co-axial high purity germanium (HPGe) gamma spectrometer (Canberra Industries) calibrated with a 20 mL ^152^Eu and ^133^Ba source. Aliquots (5–100 μL) were removed and diluted to the 20 mL standard volume for measurement at a distance of at least 15 cm from the detector until the peak area uncertainties were below 5%; dead time was kept below 4%. Spectra were analyzed using the Genie 2000 software package (Version X, Canberra Industries) using the 279 keV and 401 keV gamma lines for ^203^Pb measurement, and 238 keV and 300 keV gamma lines for ^212^Pb measurement. To determine chemical purity of the ^203^Pb and ^212^Pb elutes, 1–1.5 mL aliquots were removed and analyzed by inductively coupled plasma mass spectrometry (ICP-MS) at Chalk River Laboratories. The High-Performance Liquid Chromatography (HPLC) system used for the analysis of ^203^Pb-labeled chelators consisted of an Agilent 1260 Infinity II Quaternary Pump, Agilent 1260 autosampler, Raytest Gabi Star NaI (Tl) radiation detector, Agilent 1260 variable wavelength detector, and Agilent 1260 analytical scale fraction collector with a Phenomenex Luna 5 μm C18 100 Å liquid chromatography analytical (250 × 4.6 mm – for serum stability studies, 100 × 4.6 mm – for radiolabeling) column. RadioTLC was performed using a BioScan System 200 Image Scanner.

### Production and purification of ^228^Th nitrate

^228^Th is a by-product of the ^232^Th proton spallation used to produce ^225^Ac on TRIUMF’s main 500 MeV cyclotron (Robertson et al. 2018; Robertson et al. 2017; Robertson et al. 2020). ^228^Th is isolated from other elements during bulk thorium precipitation, as previously described (Robertson et al. 2020). To produce the ^228^Th generator stock solution, approximately 8 g of the ThCl_4_ salt was dissolved in 10 M HCl (200 mL) and loaded, via a peristaltic pump at 2 mL/min, onto a 10 mL Dowex 1 × 8 chloride form anion exchange resin column prepared as a slurry pre-conditioned with 10 M HCl (40 mL) prior to use. The column was washed with 10 M HCl (60 mL); ^228^Th did not adsorb to the column and thus was found in the load and wash fractions. Impurities were eluted from the column using 1 M HCl. The load and wash fractions were evaporated to dryness and exchanged three times with 10 M nitric acid before re-dissolving in 1 M HNO_3_ (40 mL) to produce the generator stock solution and the radionuclidic purity was assessed by gamma spectroscopy. All fractions were counted immediately after collection and were counted once again after 2 weeks to allow for the grow in of progeny (^224^Ra and ^212^Pb), which acted as a surrogate for measuring ^228^Th in gamma spectroscopy.

### The ^228^Th/^212^Pb generator principle

**Fig. 3:**
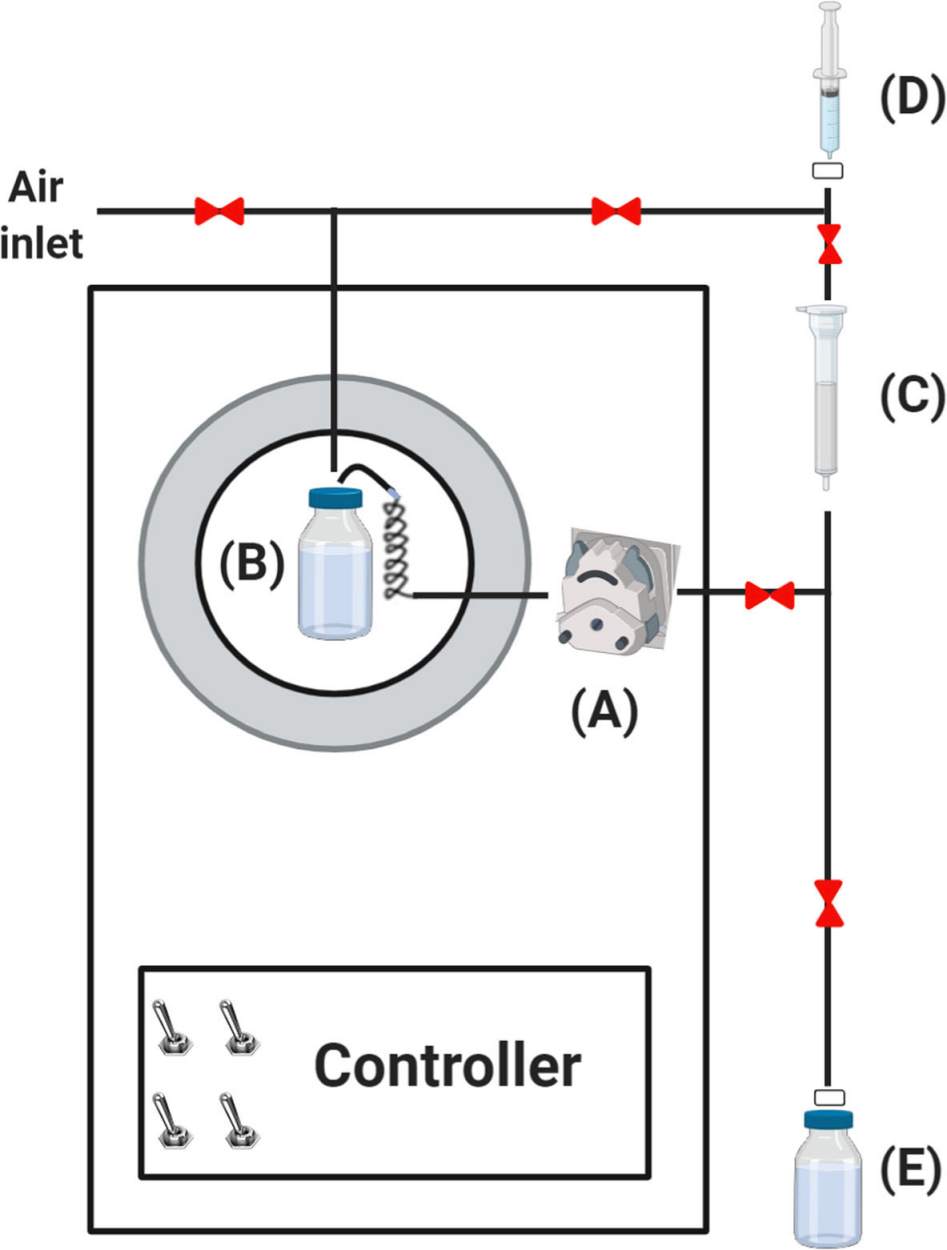
Schematic of the ^228^Th/^212^Pb generator. **a** Peristaltic pump. **b** Generator stock solution in lead shielded storage loop. **c** Pb resin column. **d** Syringe attached to a female luer fitting to control elution. **e** Collection vial for ^212^Pb(OAc)_2_. Image created with BioRender.com

The multi-component generator consists of: A peristaltic pump (Fig. 3a), running at 2 mL/min, to pump the ^228^Th generator stock solution (Fig. 3b) through an 80 mg Pb resin column (Fig. 3c), housed in a 1 mL polypropylene cartridge and preconditioned with MilliQ water (5 mL) followed by 1 M HNO_3_ (5 mL). The ^228^Th, ^224^Ra, and ^212^Bi are not retained on the column and are returned to the stock bottle via the pump using air to push the solution into a 50 mL storage loop. A syringe attached to a female luer fitting (Fig. 3d) was used to pass 1 M HNO_3_ (5 mL) through the Pb resin column to wash the column before eluting ^212^Pb with 1 M NH_4_OAc (pH 7) into a collection vial (Fig. 3e).

### Tl target production

A 1.6 mm thick, 35 mm diameter aluminum plate (6082 alloy) with a centered 10 mm diameter indent (0.25–0.30 mm depth) was used as a backing plate to hold the Tl. Natural or enriched ^203^Tl (320–330 mg) was pressed with a hydraulic press (Desktop Pellet Press, Across International, 4 MPa) into a 10 mm diameter pellet using a 10 mm die set and pressed into the aluminum backing indent (6 MPa). The target was then heated to 400 °C using a hotplate in a fume hood to allow for melting. Discolouration, indicative of thallium oxide formation was observed on the pellet surface. After cooling, the resultant thallium oxide was rinsed off the surface with deionized water. The structural integrity of the Al-Tl target was evaluated by scratch and drop testing from a height of 1.5 m onto a hard surface. The target was inspected for signs of physical damage before being vacuum sealed in a plastic envelope until irradiation.

### Target irradiation

The Tl target plate was installed in a solid target holder (Zeisler et al. 2019) and ^203^Pb produced via the ^203^Tl (p,n) ^203^Pb nuclear reaction using TRIUMF’s TR13 cyclotron (Laxdal et al. 1994). The cross section of this reaction has a threshold energy of 8 MeV and a peak at 26.5 MeV (Azzam et al. 2014), making a small energy cyclotron suitable for production. The target plate was cooled via water (back) and helium (front) during irradiation. The 13 MeV protons were degraded to ~ 12.8 MeV using a 25 μm thick aluminum foil which separated the target system from the cyclotron vacuum. The backing plate thickness was chosen to completely stop the proton beam. Irradiation was performed at 8–9 μA for 2–4 h. After irradiation, the target was left in the target holder for 18–24 h to allow short-lived radionuclides produced during the irradiation [^202m^Pb (t_1/2_ = 3.62 h)] to decay, reducing radiation exposure to personnel.

### Purification of ^203^Pb

The target was dissolved in a beaker with 2 M HNO_3_ (20 mL) on a 125 °C hot plate after which the solution was allowed to cool to ambient temperature over 1–1.5 h. A 1 mL polypropylene cartridge was packed with Pb resin (60 mg) and was conditioned with MilliQ water (5 mL) followed by 2 M HNO_3_ (5 mL). The dissolved target solution was loaded onto the column by gravity and washed with 2 M HNO_3_ (5 mL) to remove any residual thallium. The ^203^Pb was eluted with 1 M NH_4_OAc (pH 7, 3 mL) at 0.5 mL/min. The yield and radionuclidic purity of ^203^Pb in the load, wash, and elute fractions were assessed using gamma spectroscopy. Chemical purity of the ^203^Pb elute was assessed using ICP-MS to identify any stable metal species that may compete with Pb during radiolabeling reactions.

### Inductively coupled plasma mass spectrometry

Aliquots (1.5 mL; *n* = 3) of the ^212^Pb and ^203^Pb elutes were lyophilized to dryness and diluted to 10 mL using trace metal grade 1 M HNO_3_. The analysis utilized a multi-element standard^1^ for the measurement of common stable impurities which may interfere with radiolabeling. Stable impurities found in blank samples, which contained 10 mL of trace metal grade 1 M HNO_3_, were subtracted from the amount found in the elutes to quantify the impurities in the elutes.

### Non-radioactive Pb(II) complexes

Chelates (DOTA-xPy, x = 1–3) were screened for their ability to complex non-radioactive Pb(II), with metal complex formation confirmed and characterized using ESI-MS and ^1^H NMR. Briefly, Pb(OAc)_2_ (1.2 equiv., 5–6 μL of 7.8 mg/mL in H_2_O), was added to 10–15 mg of the respective chelator (1 equiv., DOTA-1Py, DOTA-2Py, DOTA-3Py), and the pH adjusted to approximately pH 6 using HCl or NaOH. The reaction mixture was stirred up to 2 h at ambient temperature, after which the solvent was evaporated in vacuo to give a white solid. [Pb(DOTA-1Py)]^−^ ESI^−^-MS: *m/z* found 642.1, calcd C_20_H_28_N_5_O_6_Pb (M^−^) 642.18. [Pb(DOTA-2Py)] ESI^+^-MS: *m/z* found 677.1, calcd C_24_H_33_N_6_O_4_Pb (M + H^+^) 677.23. [Pb(DOTA-3Py)]^+^ ESI^+^-MS: *m/z* found 710.3, calculated C_28_H_36_N_7_O_2_Pb (M^+^) 710.27. ^1^H NMR spectra of the Pb-complexes can be found in the Supporting Information.

### ^212^Pb and ^203^Pb radiolabeling studies

Chelators DOTA, TCMC, DOTA-1Py, DOTA-2Py, and DOTA-3Py were dissolved to give stock solutions (10^− 3^ M) in deionized water. Serial dilutions were used to prepare chelator solutions at 10^− 4^, 10^− 5^, and 10^− 6^ M in deionized water. A 10 μL aliquot of each chelator (or water as a negative control) was diluted with 1 M NH_4_OAc (pH 7, 80 μL). For ^203^Pb labeling studies, [^203^Pb]Pb(OAc)_2_ (50 kBq, 10 μL) was added and mixed to begin the radiolabeling reaction at ambient temperature. For ^212^Pb labeling studies, [^212^Pb]Pb(OAc)_2_ (23 kBq, 10 μL) was added to each reaction, all performed in triplicate. The iTLC plate system used for both ^203^Pb and ^212^Pb studies was iTLC-SA plates (2 cm × 10 cm, baseline at 1.5 cm) developed using EDTA (50 mM, pH 5.0). Under these conditions, the labeled Pb remained at the baseline (R_f_ = 0) and free ^203^Pb^2+^ and ^212^Pb^2+^ migrated with the solvent front (R_f_ ~ 1). Aliquots (10 μL) were removed from each reaction solution at 5, 30, and 60 min and analyzed via iTLC. For ^212^Pb, the plates were measured 24 h later to allow for the decay of free short-lived daughter products (^212^Bi, t_1/2_ = 60.55 min). ^203^Pb-complex formation with the DOTA-xPy ligands was further confirmed via analytical RP-HPLC (gradient: A, 0.1% TFA in water; B, 0.1% TFA in acetonitrile; 0–100% B over 20 min, 1 mL/min) by co-injection of pre-formed ‘stable’ Pb-complexes with the [^203^Pb]Pb-tracers and tracking both the UV and radioactive chromatograms (Figure S16).

### Human serum stability studies

To prepare the ^203^Pb labeled complexes for human serum stability studies, an aliquot (10 μL) of the 10^− 3^ M chelator solution (or water as a negative control) was added to 1 M NH_4_OAc (pH 7, 80 μL) and [^203^Pb]Pb(OAc)_2_ (100–125 kBq, 10 μL) and the reaction was allowed to proceed for 1 h at ambient temperature. Prior to the start of the study, an aliquot (10 μL) was removed and analyzed via iTLC-SA and developed as per the method described in section above, to ensure quantitative radiolabeling. Human serum (100 μL) was added and mixed and the reactions were incubated at 37 °C for 72 h post serum addition. At 8, 24, 48, and 72 h time points, an aliquot (10 μL) of the reaction mixture was removed and spotted on iTLC-SA plates and developed. At 72 h, the tubes were removed from the incubator and acetonitrile (160 μL) was added to precipitate the serum proteins. The tubes were centrifuged at 14,000 rpm for 20 min and then the supernatant was removed. The supernatant was diluted with deionized water (1.3 mL) and analyzed using analytical Radio-RP-HPLC (gradient: A, 0.1% TFA in water; B, 0.1% TFA in acetonitrile; 0–100% B over 30 min, 1 mL/min). To determine the % stability of each lead complex, the area under the curves in the radioactivity trace was calculated. The retention time of unbound “free” Pb^2+ ^was 4.3 min, [^203^Pb][Pb(DOTA)]^2−^ was 7.9 min, [^203^Pb][Pb(TCMC)]^2+^ was 9.3 min, [^203^Pb][Pb(DOTA-1Py)]^+^ was 8.6 min, [^203^Pb][Pb(DOTA-2Py)] was 9.9 min, and [^203^Pb][Pb(DOTA-3Py)]^−^ was 11.2 min.

## Results

### Isotope production

**Fig. 4:**
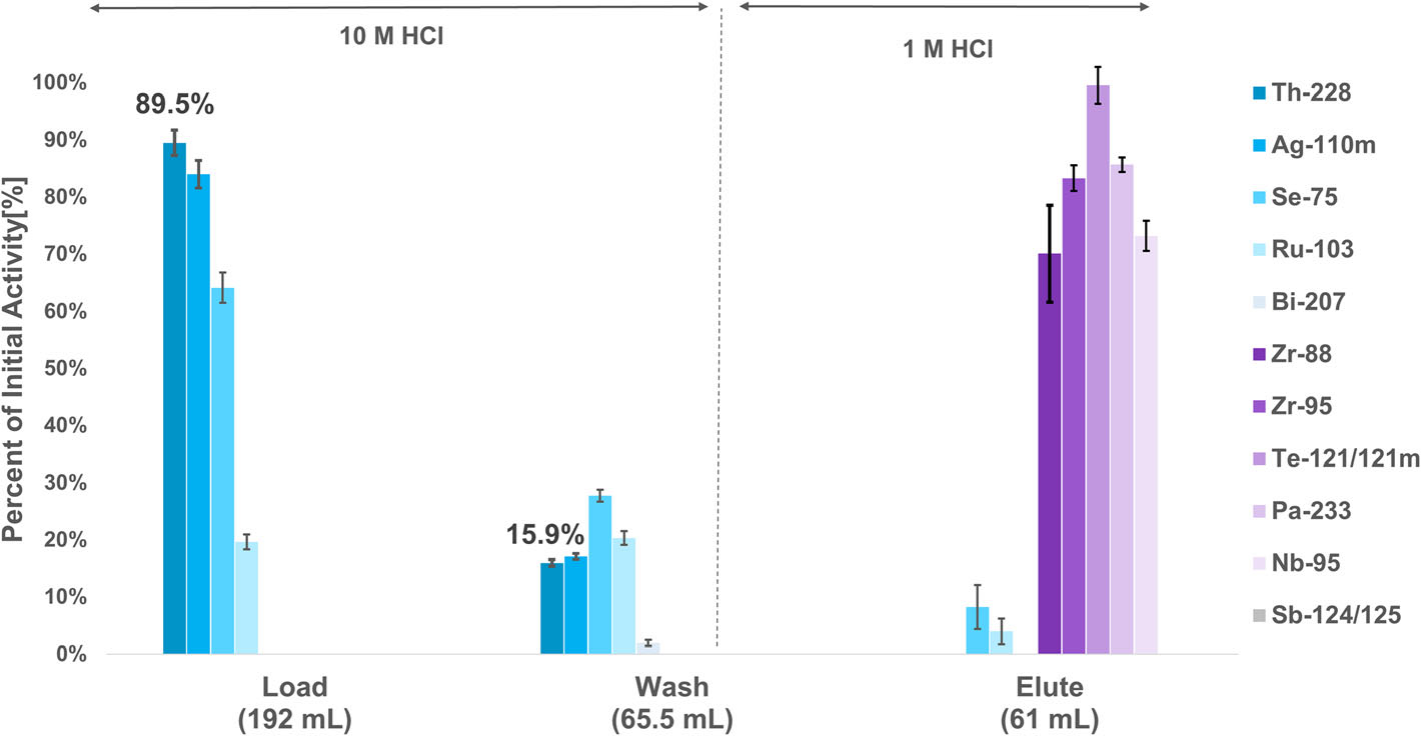
Separation of ^228^Th from other isotopes on a 1 × 8 Dowex anion exchange column (10 mL)

Initial isotopes present in the thorium precipitate included ^228^Th, ^227^Th (t_1/2_ = 18.7 d), ^75^Se (t_1/2_ = 119.8 d), ^110m^Ag (t_1/2_ = 249.8 d), ^103^Ru (t_1/2_ = 39.2 d), ^207^Bi (t_1/2_ = 31.6 y), ^88^Zr (t_1/2_ = 83.4 d), ^95^Zr (t_1/2_ = 64.0 d), ^95^Nb (t_1/2_ = 35.0 d), ^233^Pa (t_1/2_ = 26.9 d), ^121m^Te (t_1/2_ = 19.1 d), ^121^Te (t_1/2_ = 164.2 d), ^124^Sb (t_1/2_ = 60.2 d), and ^125^Sb (t_1/2_ = 2.8 y) (Fig. 4**)**. Further information on the activity of each isotope can be found in Table S3. ^95^Nb and ^95^Zr were removed via anion exchange, giving load and wash fractions for the generator stock solution containing ^75^Se,^110m^Ag, ^103^Ru, ^207^Bi, and ^227^Th, in addition to ^228^Th.

**Fig. 5:**
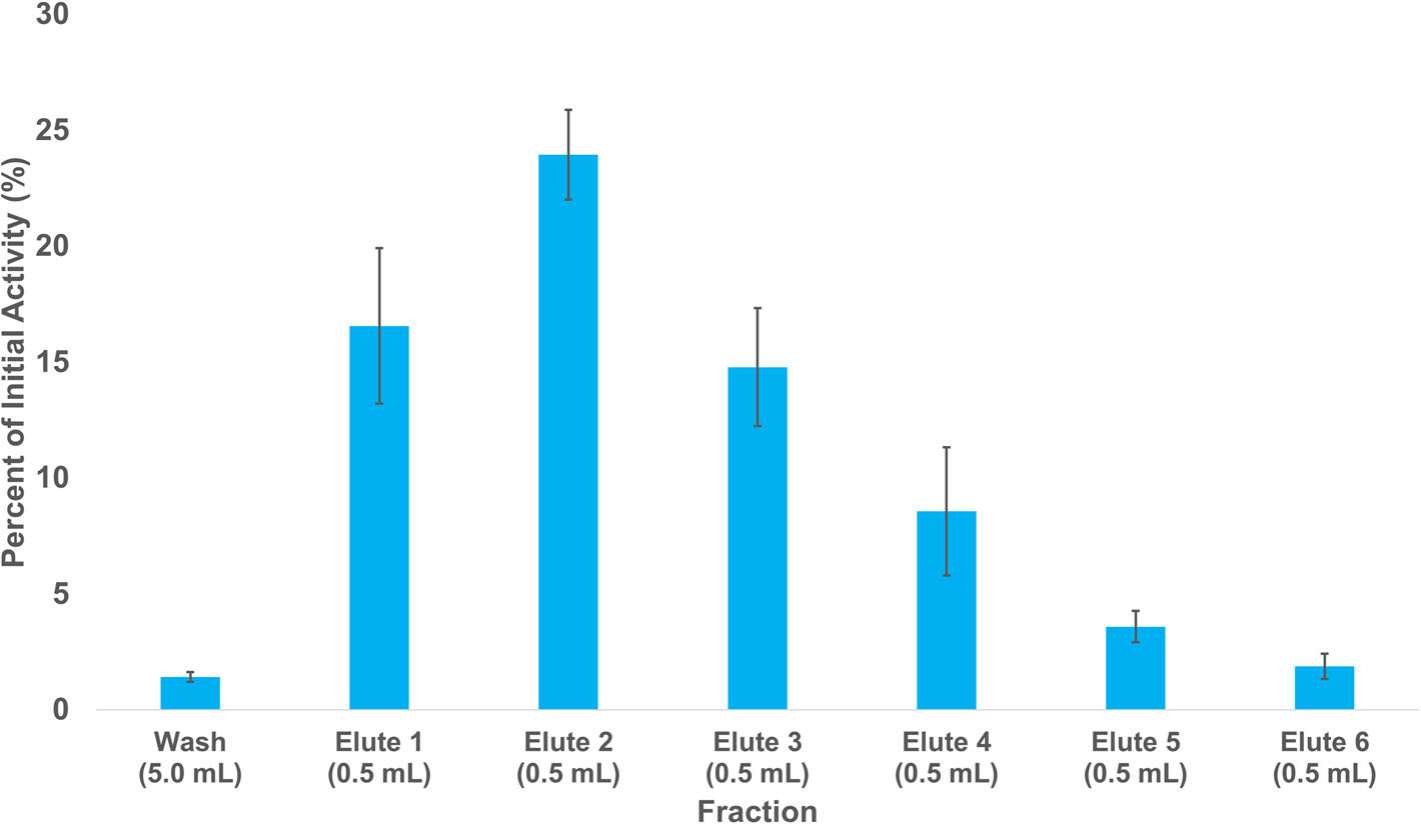
Average elution profile for ^228^Th/^212^Pb separation from Pb resin using NH_4_OAc (1 M, pH 7) (*n* = 4)

The purification of ^212^Pb from its parent isotope ^228^Th with a generator based on Pb-selective extraction resin produced product with > 99% radiochemical purity (Figure S1) with an average yield of 69.3 ± 4.4%. The initial radionuclide generator solution, which initially contained 9.780 ± 0.002 MBq of ^228^Th, was used to supply ^212^Pb for at least 2 years. The average elution profile is shown in Fig. 5.

**Fig. 6:**
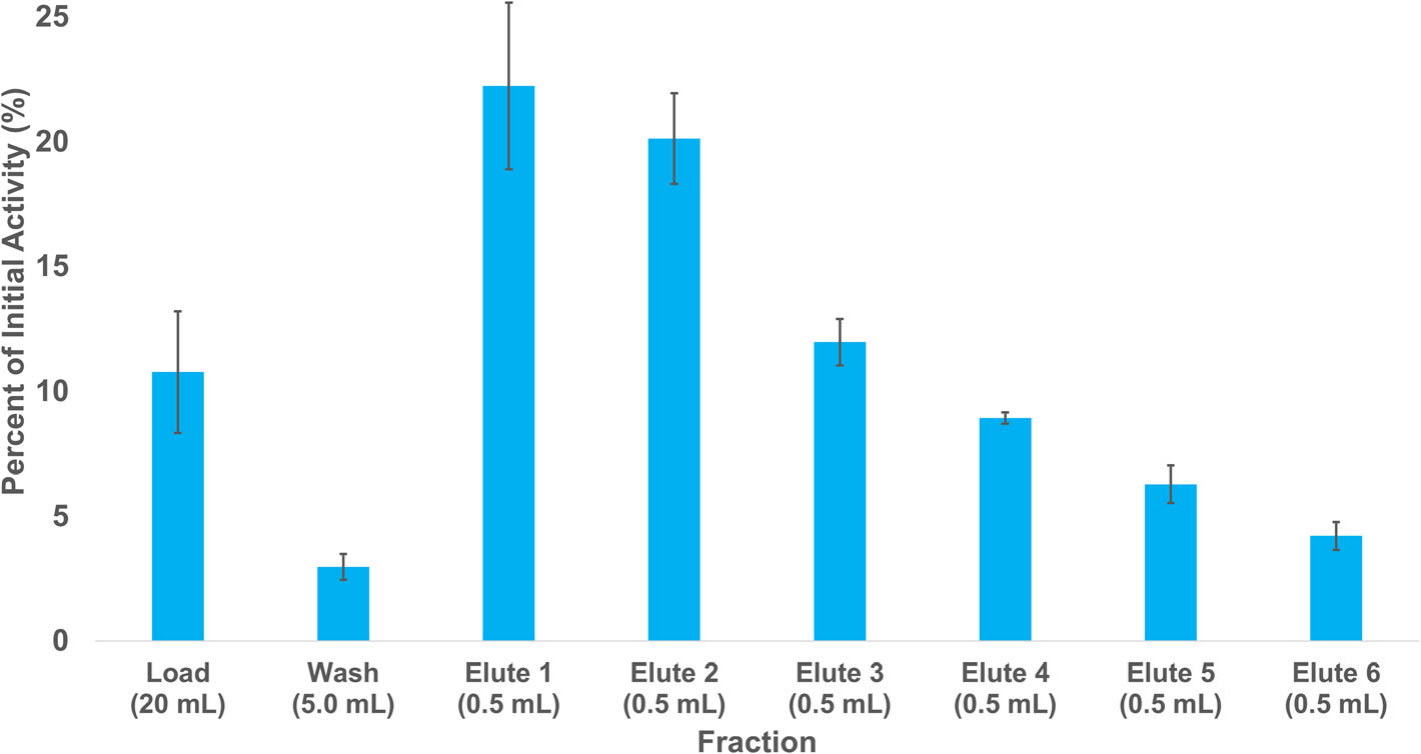
Average elution profile for ^203^Pb purification from Pb resin using NH_4_OAc (1 M, pH 7) (*n* = 6)

Radiochemically-pure ^203^Pb was produced and isolated from irradiated aluminum-backed thallium targets (Figure S2) with an average yield, decay corrected to the end of beam (EOB), of 73.8 ± 2.1% (*n* = 3) and all yields correspond to a calculated saturation yield of 134 ± 25 MBq/μA. Natural Tl targets irradiated for 2 h at 8 or 9 μA produced, on average as determined by gamma spectroscopy, 27.3 ± 4.7 MBq (*n* = 5) and 32.9 ± 2.7 MBq (*n* = 8) of ^203^Pb, respectively. Enriched ^203^Tl targets irradiated for 3.5 to 4 h at a current of 8 μA produced, as determined by gamma spectroscopy, 175.3 MBq and 201.9 MBq of ^203^Pb, respectively, corresponding to a calculated saturation yield of 483 ± 3 MBq/μA (*n* = 2). The elution profile of an average ^203^Pb separation is shown in Fig. 6.

Although both Pb products were observed to be radionuclidically pure, the elemental purity was assessed via ICP-MS to quantify any stable impurities that may have interfered with radiolabeling (Table 1). In the ^203^Pb product, moderate values of Tl were observed with 58,220 ± 35,392 ppb (175 ± 105 μg; *n* = 3) present in the entire elute. Although there was a ~ 1700 fold reduction in the mass of the Tl found in the elute compared to the mass of the Tl present initially in the target, an additional washing step may help to further reduce the mass found in the elute, which will be necessary for clinical applications. Tl, Ca, Ge, Ni, and Zn were not found to be at levels above the blank concentration in the ^212^Pb product, while Th and Ti were not detected in the ^203^Pb product. Mg, Al, Ca, Fe, Co, Ni, Cu, Zn, and Pb were found in low to modest amounts. Ca, Al, and stable Pb were of greatest concern as DOTA has a strong affinity for Ca and Al (Clarke and Martell 1991), while stable Pb can compete with radioactive Pb during the radiolabeling process as they are chemically identical and cannot be separated. In the ^212^Pb product, the concentration of stable Pb was lower (2.1 ± 2.0 ppb; 6.4 ± 6.0 ng) than in the ^203^Pb product (495 ± 218 ppb; 1.49 ± 0.66 μg); however, Mg and Ti concentrations were higher at 612 ± 226 ppb and 354 ± 168 ppb, respectively. ICP-MS results for additional elements can be found in Table S1.

**Table 1:** Metal content in elute fractions in ppb (μg/L) determined by ICP-MS (*n* = 3)

**Isotope**	**Mg**	**Al**	**Ca**	**Ti**	**Fe**	**Co**
^**203**^**Pb**	44 ± 14	168 ± 152	568 ± 263	N.S.	18 ± 11	0.3 ± 0.5
^**212**^**Pb**	612 ± 226	22 ± 9	N.S.	354 ± 168	N.S.	26 ± 11
**Isotope**	**Ni**	**Cu**	**Zn**	**Tl**	**Pb**	**Th**
^**203**^**Pb**	10 ± 11	3 ± 2	21 ± 4	58,220 ± 35,392	495 ± 218	N.S.
^**212**^**Pb**	N.S.	3 ± 2	N.S.	N.S.	2 ± 2	24,352 ± 16,227

*N.S.* Not significant

### Radiolabeling studies

All radiolabeling reactions in this study were performed at room temperature and pH 7 in triplicate and the percent radiochemical yield (% RCY) is reported at the one-hour time point as determined by radio-iTLC. The gold standard for Pb^2+^ complexation, *p*-SCN-Bn-TCMC, had ^203^Pb-radiochemical yields of 97.2 ± 0.6%, 96.9 ± 0.6%, 43.7 ± 1.0%, and 3.7 ± 0.4% (*n* = 3), at concentrations of 10^− 4^ to 10^− 7^ M, respectively (Fig. 6). When the study was repeated with ^212^Pb, the RCYs were 97.8 ± 0.4%, 98.1 ± 0.5%, 80.8 ± 8.9%, and 13.9 ± 1.6% (*n* = 3), respectively. DOTA, although not the gold standard for Pb complexation but used in a number of ^212^Pb/^203^Pb preclinical studies (Bartoś et al. 2013; Li et al. 2017; Garmestani et al. 2005; Miao et al. 2005; Jianquan et al. 2017; Miao et al. 2008), was able to efficiently complex ^203^Pb with radiochemical yields of 96.1 ± 1.0%, 75.8 ± 9.4%, 3.0 ± 0.8%, and 1.5 ± 0.2% at concentrations of 10^− 4^ M to 10^− 7^ M, respectively. When the study was repeated with ^212^Pb, the RCYs were 97.6 ± 0.1%, 96.5 ± 0.8%, 4.3 ± 1.2%, and 3.6 ± 0.5%, respectively.

**Fig. 7:**
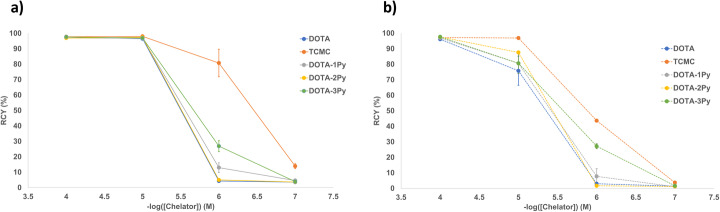
Radiochemical yield (RCY, %) for **a** ^212^Pb and **b** ^203^Pb radiolabeling reactions at pH 7 and room temperature at 1 h at chelator concentrations of 10^− 4^ – 10^− 7^ M

With 1–3 pyridine rings in place of the carboxylic acid groups found on DOTA, the chelators DOTA-1Py, DOTA-2Py, and DOTA-3Py were able to efficiently complex ^203^Pb at the 10^− 4^ M concentration (RCYs of 97.0 ± 0.3, 97.6 ± 0.3%, and 97.7 ± 0.5%, respectively). When repeated with ^212^Pb, all three of the chelators were able to complex ^212^Pb efficiently at not only 10^− 4^ M, but also at 10^− 5^ M. For both ^203^Pb and ^212^Pb, the RCYs reduced sequentially at lower concentrations of 10^− 5^ to 10^− 7^ M. DOTA-1Py had ^203^Pb-RCYs of 80.6 ± 6.6%, 7.7 ± 5.0%, and 1.1 ± 0.2% at concentrations of 10^− 5^ to 10^− 7^ M, respectively, and when repeated with ^212^Pb over concentrations of 10^− 4^ to 10^− 7^ M, the RCYs were 97.0 ± 0.9%, 96.8 ± 0.6%, 13.0 ± 3.1%, and 4.6 ± 1.1%, respectively. DOTA-2Py had ^203^Pb-RCYs of 87.6 ± 0.3%, 1.7 ± 0.6%, and 1.2 ± 0.3% at concentrations of 10^− 5^ to 10^− 7^ M, respectively. When repeated with ^212^Pb, at concentrations of 10^− 4^ to 10^− 7^ M, the RCYs were 96.9 ± 0.8%, 97.4 ± 0.6%, 5.0 ± 0.4%, and 3.7 ± 0.8%, respectively. DOTA-3Py had ^203^Pb-RCYs of 80.6 ± 6.6%, 27.2 ± 1.56%, and 1.7 ± 0.1% at concentrations of 10^− 5^ to 10^− 7^ M, respectively. When repeated with ^212^Pb, with concentrations of 10^− 4^ to 10^− 7^ M, the RCYs were 97.6 ± 0.7%, 97.0 ± 0.1%, 26.9 ± 3.6%, and 3.9 + 0.9%, respectively. The ^212^Pb and ^203^Pb radiolabeling results of these chelators are shown in Fig. 7. Metal complexation with the chelators was also confirmed by synthesizing non-radioactive Pb-complexes of all chelates and characterizing by mass spectrometry and ^1^H NMR spectroscopy. The distinct isotope distribution pattern for Pb (^204^Pb [1.4%], ^216^Pb [24.1%], ^207^Pb [22.1%], ^208^Pb [52.1%]) in the MS helped to confirm metal complexes (Figures S6, S7, S8, S9, S10).

### Human serum stability studies

The stability of ^203^Pb complexes in human serum for [^203^Pb][Pb(TCMC)]^2+^, [^203^Pb][Pb(DOTA]^2−^, [^203^Pb][Pb(DOTA-1Py)]^−^, [^203^Pb][Pb(DOTA-2Py)], [^203^Pb][Pb(DOTA-3Py)]^+^ is shown in Table 2. The radio-HPLC traces of the human serum stability reactions are shown in Figure S14 in the supplementary data. All of the commercial and pyridine-based chelators were exceptionally stable (> 95% stability at 72 h), as shown by both iTLC and radio-HPLC (Figure S15).

**Table 2:** In vitro stability of ^203^Pb-labeled chelator complexes at 37 °C in human serum (*n* = 3)

^203^Pb-complex % stable	Time point			
	8 h	24 h	48 h	72 h
[^203^Pb][Pb(TCMC)]^2+^	98.0 ± 0.5%	98.1 ± 0.2%	98.2 ± 0.3%	97.2 ± 0.7%
[^203^Pb][Pb(DOTA)]^2−^	97.1 ± 0.7%	97.3 ± 0.6%	98.1 ± 0.2%	97.4 ± 0.5%
[^203^Pb][Pb(DOTA-1Py)]^−^	97.4 ± 0.4%	97.7 ± 0.3%	98.1 ± 0.4%	97.5 ± 0.7%
[^203^Pb][Pb(DOTA-2Py)]	97.7 ± 0.1%	97.8 ± 0.1%	97.8 ± 0.9%	97.1 ± 0.2%
[^203^Pb][Pb(DOTA-3Py)]^+^	98.4 ± 0.3%	98.0 ± 0.4%	98.4 ± 0.1%	97.7 ± 0.3%
Negative Control	1.2 ± 0.3%	1.4 ± 0.2%	1.4 ± 0.2%	1.5 ± 0.2%

## Discussion

^203^Pb and ^212^Pb produced at TRIUMF, either on the TR13 cyclotron or as a by-product of 500 MeV proton irradiation of ^232^Th, respectively, can be rapidly separated (< 3 h for ^203^Pb and < 1 h for ^212^Pb) with high radionuclidic purity (> 99%), moderate yield (73.8 ± 2.1% for ^203^Pb, 69.3 ± 4.4% for ^212^Pb), and a chemical purity that is suitable for pre-clinical screening of potential chelators to be used for theranostic purposes.

The direct elution of the Pb products into a radiolabeling compatible solution (1 M NH_4_OAc, pH 7) reduces the number of steps in the purification procedure and allows for rapid, immediate use of the product for potential clinical purposes, an advantage over methods that require the use of several columns or solution exchange, which can prolong the radiochemist’s exposure to radiation. The use of a single Pb-selective extraction resin allows for easy separation of ^203^Pb when the Tl target is dissolved in 2 M HNO_3_, Pb readily sorbs onto the resin while thallium passes through; thus there is no need for solution exchange to produce a column compatible loading solution, reducing the length of the procedure. In addition, when the nitric acid concentration is 2 M the capacity factor of Pb (k’_Pb_) is nearly 100 times greater than that of thallium (k’_Tl_) (Philip Horwitz et al. 1994). Due to the high capacity factor of Pb on this resin, a single 60 mg Pb resin column allowed for a 1700 fold reduction in Tl content, which is of importance for clinical purposes due to the high toxicity of Tl. As shown in Table 1, in the entire 3 mL elute 175 (+ 105) μg of thallium was found, and although the mass is below regulated toxicity levels (occupational limit of 0.1 mg/m^3^ = approx. 6.5 mg of thallium assuming an average body mass of 65 kg) (Kemnic and Coleman 2020), it would be ideal to further reduce Tl content to reduce potential radiolabeling interference.

During method development, we found that increasing the column mass from 30 to 60 mg reduced the percent of initial activity lost in the load from 40.3 + 1.0% to 20.5 + 0.5% (Figure S3). Further increasing the Pb-resin mass did not improve activity losses in the load fraction. The volume of the loading solution was then optimised, and it was found that reducing the loading volume from 30 to 20 mL reduced the loss of activity in the load fraction from 20.5 + 0.5% to 8.7 + 0.3% (Figure S4). Other investigators have loaded uncooled solutions onto columns (Li et al. 2017), however, in this study cooling the solution to ambient temperature prior to loading the column was found to be critical, as it was found that the average yield of eluted ^203^Pb dropped to 36.1 + 9.6% at elevated solution temperatures when compared to 73.8 + 2.1% cooled (Figure S5); we hypothesize that the reduction in yield in the former was likely due to damage caused to the resin’s structure upon exposure to the elevated temperature.

Previous animal studies have utilized approximately 6–7.5 MBq of ^203^Pb-labeled bioconjugate for imaging studies (Miao et al. 2008; Yang et al. 2019). In human studies by dos Santos and colleagues, 250–310 MBq of ^203^Pb-labeled bioconjugate were required for imaging and it was estimated that up to 750 MBq could be utilized in future dosimetry studies (dos Santos et al. 2019). With 201.9 MBq of ^203^Pb produced by irradiating enriched Tl at 8 μA for 4 h, it is reasonable to expect that with greater beam current and longer irradiation times that sufficient quantities of ^203^Pb can be produced to enable both preclinical and clinical studies.

Despite the use of high purity (99.99% metals basis) thallium, significant levels of stable Pb (1.49 + 0.66 μg) were found in the elute. At this level, experiments utilizing lower amounts of activity will need to carefully consider the impact of molar activity for radiolabeling and in vitro studies. Longer irradiation times and higher beam current will produce more ^203^Pb and thus further decrease this ratio. Radiolabeling results would be most improved if the source of the stable Pb was identified and the mass reduced. A potential source of stable Pb may be the thallium metal used for target manufacturing and future studies will evaluate different methods to increase molar activity.

Small (20 mCi) ^224^Ra generators are available, but their production is reliant on the extraction of ^228^Th from an aging stockpile of ^232^U (Nuclear Science Advisory Commitee : Isotopes Subcommmitee 2015). Due to the comparatively short half life of ^224^Ra (t_1/2_ = 3.63 d), a ^224^Ra/^212^Pb generator can only be used for 1–2 weeks, thus increasing costs of isotope production. ^228^Th/^212^Pb generators, however, could potentially be used for extended periods of time. Herein we have reported the production and isolation of ^228^Th as a by-product of the proton irradiation of ^232^Th on TRIUMF’s 500 MeV cyclotron and is isolated by peroxide-induced precipitation (Robertson et al. 2020). Previous generators used cation exchange columns and eluted ^212^Pb with water (Zucchini and Friedman 1982), which is not immediately radiolabeling compatible, while others utilized [^228^Th] barium stearate and collected ^212^Pb on glass walls (Hassfjell and Hoff 1994) and with bubblers (Hassfjell 2001), and although these generators were effective, scaled-up production would be challenging, unlike the generator introduced in these studies. Although there were radiochemical impurities in the final generator stock solution, as shown in Fig. 4, with the high selectivity of the Pb resin employed in the generator, none of these contaminants were observed in the elute fraction. The elute was also found to be low in stable Pb (6 + 6 ng in 3 mL), which resulted in higher radiolabeling yields, as observed in Fig. 6, for all chelators, thus further demonstrating the potential of the pyridine-based cyclen analogues (DOTA-xPy, x = 1–3) for chelation of Pb isotopes.

In previous animal studies, 0.2–7.4 MBq of ^212^Pb-labeled bioconjugates were used for biodistribution and therapy studies (Miao et al. 2005; Stallons et al. 2019). In human studies, dos Santos and colleagues predict the dose range for ^212^Pb-labeled bioconjugates to be 50 to 150 MBq (dos Santos et al. 2019). Upon assembly, the generator produced 9.780 + 0.002 MBq of ^228^Th and provided ~ 10 MBq of ^212^Pb on elution, capable of enabling both preclinical biodistribution and therapy studies. Although the current activity produced is not high enough for clinical applications, further scale up efforts are underway. The current purification procedure gives modest ^212^Pb yields of 69.3 + 4.4%, which may be due to the presence of approximately 8 g of ^232^Th present in the generator stock solution hindering the sorption of ^212^Pb to the column. The ideal resin mass used for the purification was 80 mg, as opposed to 60 mg for ^203^Pb, as it was found that larger masses did not increase the yield, but did increase the elute volume required to reach the same yield. The mass of Th found in the elute (24,352 ± 16,227 ppb, 73.1 + 48.6 μg) represents a separation factor of approximately 10^5^ (see Table 1). Future work will examine process optimization that may further reduced Th burden.

All chelators showed quantitative RCYs (> 95%) at room temperature for both ^203^Pb and ^212^Pb at a concentration of 10^− 4^ M. However, at 10^− 5^ M the ^212^Pb radiolabeling yield was higher for all tested chelators and bioconjugates, demonstrating a positive effect of increased molar activity of the ^212^Pb compared to ^203^Pb. In order to avoid the accumulation of stable ^208^Pb, the terminus of the ^212^Pb decay chain, the generator was milked 24 h prior to radiolabeling tests and the next day’s elute was used for labeling immediately in order to minimize the grow in of the stable daughter. In addition to the commercial chelators, DOTA and *p*-SCN-Bn-TCMC, three pyridine-based cyclen ligands, DOTA-1Py, DOTA-2Py, and DOTA-3Py, were also screened for their ability to complex ^203^Pb/^212^Pb, as the softer pyridine (N-) donors were hypothesized to form stable metal-ligand coordinate bonds with the softer Pb^II^ ion (Price and Orvig 2014; Ramogida and Orvig 2013). At 10^− 5^ M and ambient temperature, it was observed that for the pyridine-based chelators, as the number of pyridine groups increased, so did the radiolabeling yield; this trend was observed for both ^212^Pb and ^203^Pb. With the greatest radiolabeling yield of the pyridine-based chelators and with high human serum stability (> 97% at 72 h), DOTA-3Py shows the greatest promise as a new Pb chelator and is a good candidate for incorporation into a bioconjugate for theranostic purposes.

## Conclusion

Routine production of both members of the ^212^Pb/^203^Pb theranostic pair was established at TRIUMF. ^228^Th, a by-product of ^232^Th spallation on TRIUMF’s 500 MeV cyclotron, was used to produce a novel ^228^Th/^212^Pb generator and was combined with ^203^Pb production via thallium irradiation with 13 MeV protons. Both ^203^Pb and ^212^Pb were produced at quantities and purities (radionuclidic and chemical) acceptable for preclinical radiopharmaceutical screening. Increased irradiation times may lead to production at clinical quantities. Separation of the lead products was achieved using a Pb-selective extraction chromatographic resin in moderate yields (73.8 + 2.1% for ^203^Pb, 69.3 + 4.4% for ^212^Pb) in a form suitable for direct radiolabeling. The lead products were used to screen the radiolabeling ability and serum stability with commercially available (DOTA and *p*-SCN-Bn-TCMC) and pyridine-based DOTA derivative chelators (DOTA-1Py, DOTA-2Py, and DOTA-3Py). DOTA-1Py, −2Py, and -3Py all exhibited ability to complex ^212^Pb/^203^Pb at ambient temperature, with [^212^Pb/^203^Pb]Pb-DOTA-3Py showing the highest radiolabeling yield of the three. Further investigation of the Pb^II^-coordination chemistry with these ligands as well as preparation of bioconjugates for in vivo studies are planned in the future. In conclusion, together these studies demonstrate the ability of TRIUMF to produce a theranostic pair that can be used for pre-screening potential radiopharmaceuticals at the pre-clinical level with the potential to increase production to the clinical level.

## Supplementary Information

**Additional file 1.** Detailed ICP-MS results, activities of the components in the thorium precipitate solution, representative gamma spectra for ^203^Pb and ^212^Pb, elution profiles for radiochemical purification method development runs, MS and ^1^H NMR of Pb-complexes, representative radio-HPLC chromatograms of [^203^Pb]Pb-complexes and human serum stability studies, representative radio-iTLC chromatograms of ^203^Pb- and ^212^Pb-radiolabeling.

## Data Availability

The datasets used and/or analyzed during the current study are available from the corresponding author(s) on reasonable request.

## Footnotes

1 Na, Mg, Al, Ca, Ti, V, Cr, Mn, Fe, Ni, Co, Cu, Zn, Ga, Ge, As, Se, Rb, Sr, Zr, Nb, Mo, Ru, Rh, Pd, Ag, Cd, Sn, Te, Cs, Ba, La, Ce, Pr, Nd, Sm, Eu, Gd, Dy, Ho, Er, Tm, Yb, Lu, Hf, Ta, W, Re, Os, Ir, Pt, Au, Tl, Pb, Th, Hg
