# Supplementary material for: Production, purification, and radiolabeling of the 203Pb/212Pb theranostic pair
Source: EJNMMI Radiopharm Chem. 2021 Feb 1;6:6. doi: 10.1186/s41181-021-00121-4 (PMC7851237; doi:10.1186/s41181-021-00121-4)
Supplement: Supplementary file 1 — Additional file 1. Detailed ICP-MS results, activities of the components in the thorium precipitate solution, representative gamma spectra for 203Pb and 212Pb, elution profiles for radiochemical purification method development runs, MS and 1H NMR of Pb-complexes, representative radio-HPLC chromatograms of [203Pb]Pb-complexes and human serum stability studies, representative radio-iTLC chromatograms of 203Pb- and 212Pb-radiolabeling. [file 41181_2021_121_MOESM1_ESM.pdf]

Supporting Information

Production, Purification, and Radiolabeling of the $^{203}Pb/^{212}Pb$ Theranostic Pair

Brooke L. McNeil[1,2†], Andrew K. H. Robertson[1,3†], Winnie Fu[1], Hua Yang[1], Cornelia Hoehr[1], Caterina F. Ramogida[1,2*], Paul Schaffer[1,2,4*]

[1]Life Sciences Division, TRIUMF, Vancouver BC, Canada.
[2]Department of Chemistry, Simon Fraser University, Burnaby BC, Canada.
[3]Department of Physics and Astronomy, University of British Columbia, Vancouver BC, Canada.
[4]Department of Radiology, University of British Columbia, Vancouver BC, Canada.
[†]Both authors provided equal contribution to this work.

*Correspondence: cfr@sfu.ca; pschaffer@triumf.ca

Table S1. Detailed ICP-MS results. Trace metal content in ^{203}Pb and ^{212}Pb elute in ppb (µg/L) (*n* = 3). N.S. = Not significant.

Isotope	Mg	Al	Ca	Ti	Fe	Co	Ni	Cu	Zn
^{203}Pb	44 ± 14	168 ± 152	568 ± 263	N.S.	18 ± 11	0.3 ± 0.5	10 ± 11	2.7 ± 1.8	21 ± 4
^{212}Pb	612 ± 226	22 ± 9	N.S.	354 ± 168	N.S.	26 ± 11	N.S.	3.1 ± 2.1	N.S.

Isotope	Tl	Pb	Th	Cr	Mn	Sr	Mo	Sn
^{203}Pb	58220 ± 35392	495 ± 218	N.S	0.2 ± 0.1	0.4 ± 0.1	0.9 ± 0.5	0.1 ± 0.1	0.1 ± 0.1
^{212}Pb	N.S.	2.1 ± 2.0	24352 ± 16227	1.4 ± 1.9	0.3 ± 0.3	10 ± 3	0.3 ± 0.3	0.1 ± 0.0

Table S2. Trace metal content in ^{203}Pb and ^{212}Pb elute in ng (*n* = 3)

Isotope	Mg	Al	Ca	Ti	Fe	Co	Ni	Cu	Zn
^{203}Pb	132 ± 42	503 ± 456	1703 ± 789	N.S.	54 ± 33	0.9 ± 1.4	31 ± 33	8.0 ± 5.5	64 ± 11
^{212}Pb	1837 ± 677	67 ± 26	N.S.	1063 ± 505	N.S.	77 ± 33	N.S.	9.3 ± 6.2	N.S.

Isotope	Tl	Pb	Th	Cr	Mn	Sr	Mo	Sn
^{203}Pb	174659 ± 106175	1486 ± 655	N.S.	0.7 ± 0.3	1.1 ± 0.4	2.6 ± 1.6	0.4 ± 0.2	0.3 ± 0.2
^{212}Pb	N.S.	6.4 ± 6.0	73055 ± 48681	4.2 ± 5.7	1.0 ± 0.8	30 ± 10	0.8 ± 0.8	0.4 ± 0.1

Table S3. Activity of components of the initial thorium precipitate solution (192 mL).

Radionuclide	Activity + Uncertainty (Bq)
^{75}Se	$2.04x10^5 \pm 6.32x10^3$
^{88}Zr	$1.45x10^5 \pm 1.63x10^4$
^{95}Nb	$2.64x10^7 \pm 6.68x10^5$
^{95}Zr	$2.08x10^7 \pm 3.94x10^5$
^{103}Ru	$6.19x10^5 \pm 2.84x10^4$
^{110m}Ag	$5.20x10^5 \pm 1.27x10^4$
^{121}Te	$1.66x10^6 \pm 5.13x10^4$
^{121m}Te	$1.74x10^6 \pm 5.37x10^4$
^{124}Sb	$3.58x10^6 \pm 8.96x10^4$
^{125}Sb	$1.75x10^6 \pm 3.88x10^4$
^{207}Bi	$2.30x10^5 \pm 6.72x10^3$
^{227}Th	$1.06x10^6 \pm 3.27x10^4$
^{233}Pa	$1.08x10^7 \pm 1.18x10^5$
^{228}Th	$9.78x10^6 \pm 5.00x10^2$

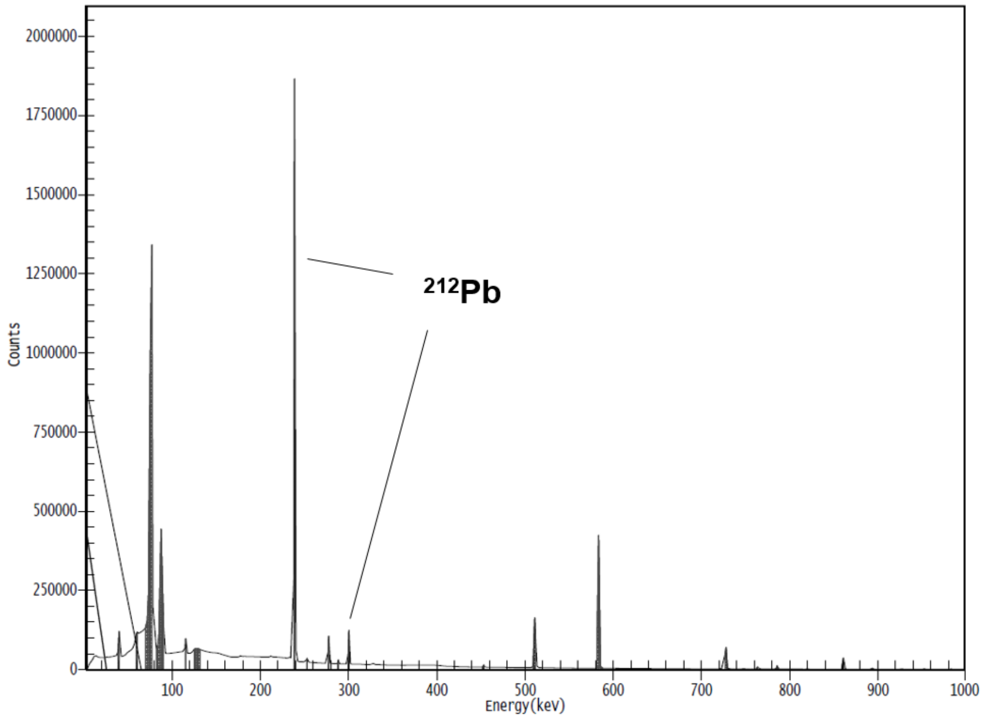

Figure S1. Gamma spectrum of the ^{212}Pb elute. Unlabelled peaks correspond to progeny (^{208}Tl at 510.8 and 583.1 keV, ^{212}Bi at 727.3 keV).

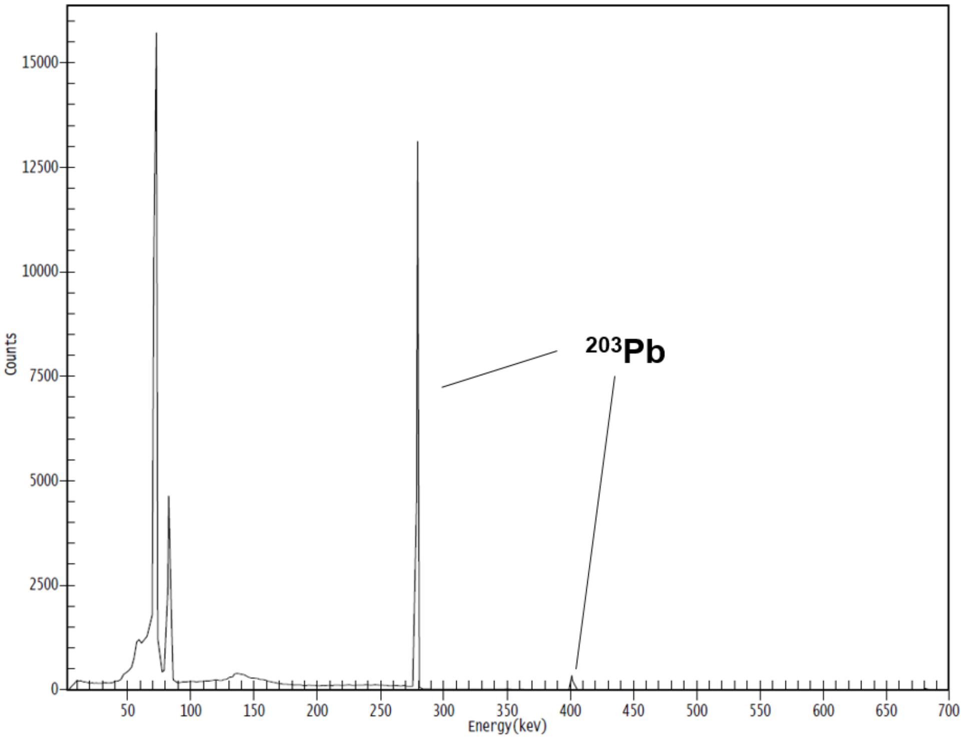

Figure S2. Gamma spectrum of the ^{203}Pb elute.

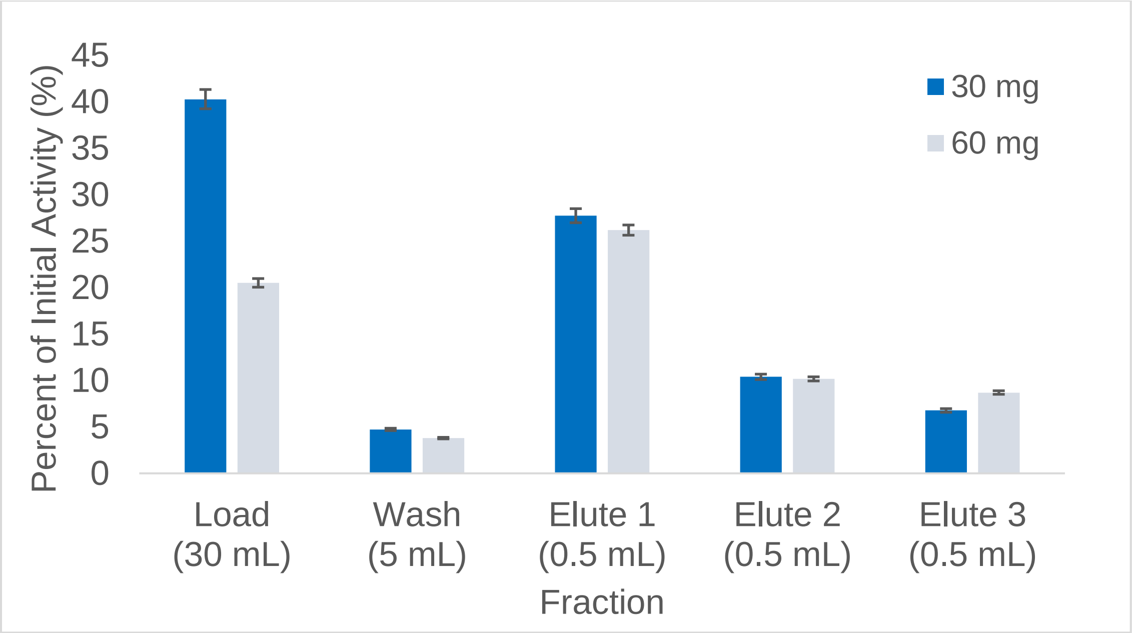

Figure S3. Effect of Pb resin mass (30 or 60 mg) on the ^{203}Pb elution profile.

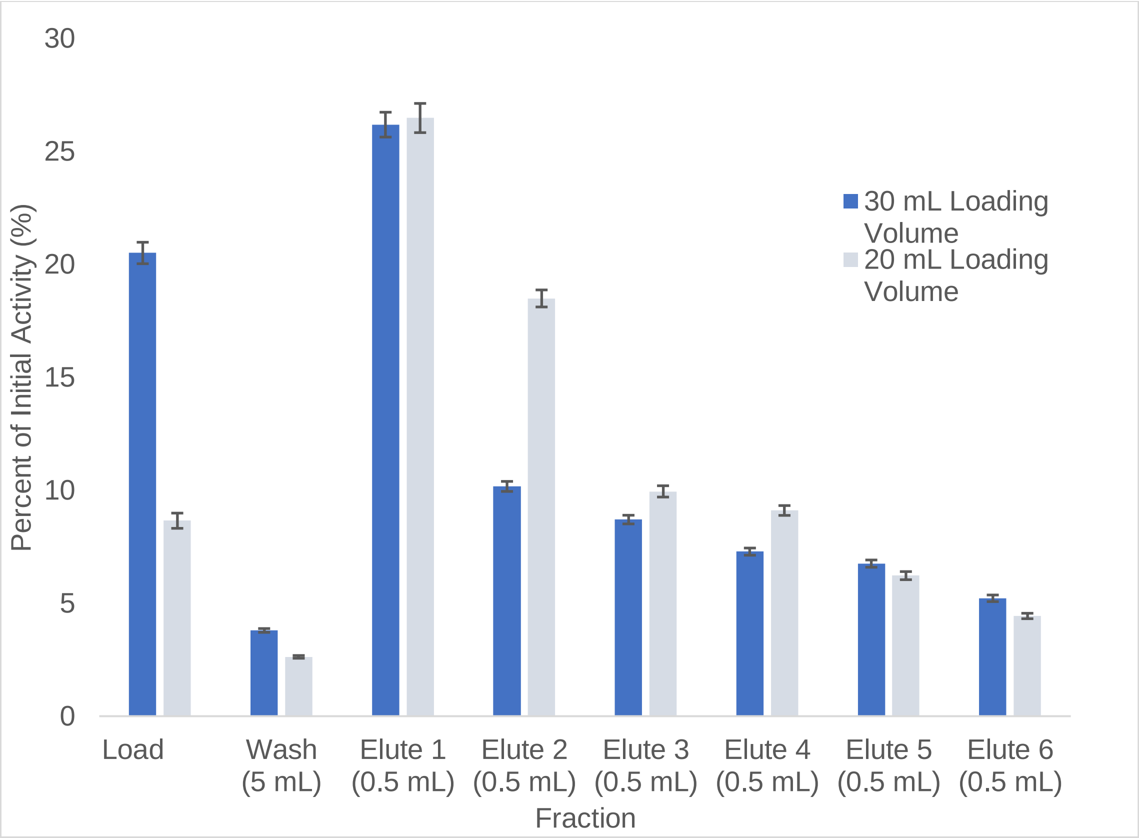

Figure S4. Effect of loading volume on the elution profile of ^{203}Pb (on a 60 mg Pb resin).

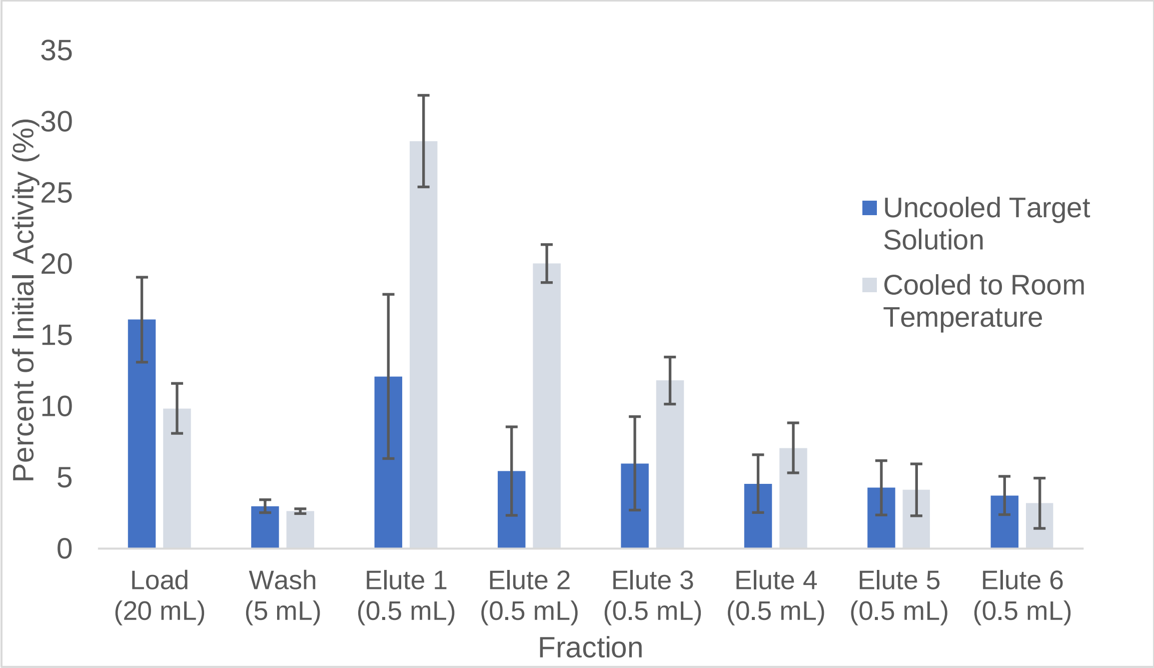

Figure S5. Effect of temperature on the elution profile of ^{203}Pb (using a 60 mg Pb resin).

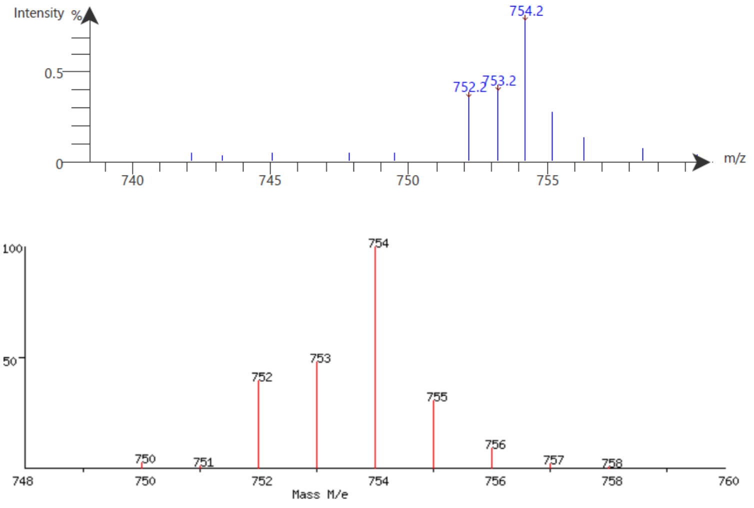

Figure S6. Experimental (top) and theoretical (bottom) mass spectrum of $[Pb(TCMC)]^{2+}$ (*m/z* calcd $(M-H])^{+}$ = 745.2).

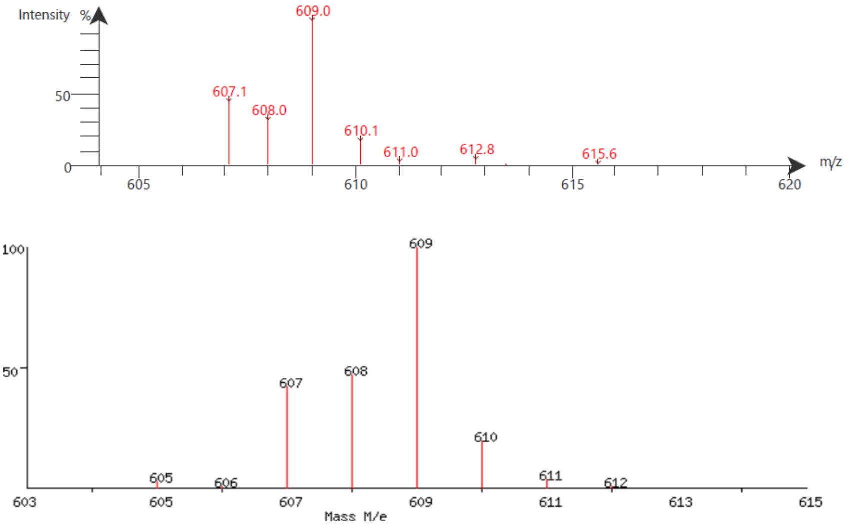

Figure S7. Experimental (top) and theoretical (bottom) mass spectrum of $[Pb(DOTA)]^{2-}$ (*m/z* calcd $(M+H)^{-}$ = 609.1).

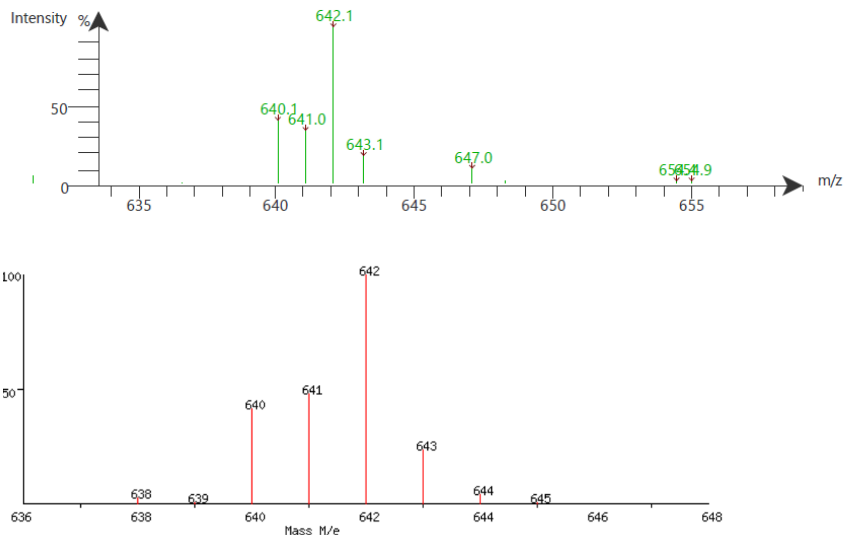

Figure S8. Experimental (top) and theoretical (bottom) mass spectrum of $[Pb(DOTA\text{-}1Py)]^-$ (*m/z* calcd $(M)^-$ = 642.2).

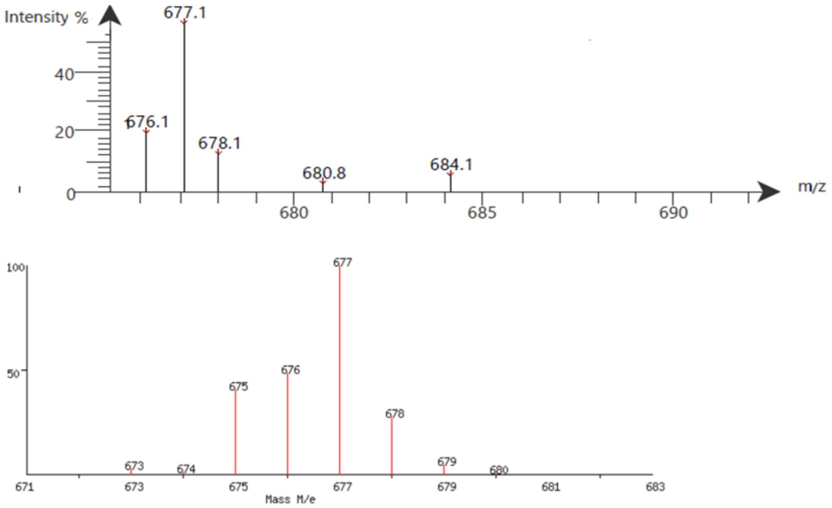

Figure S9. Experimental (top) and theoretical (bottom) mass spectrum of [Pb(DOTA-2Py)] (*m/z* calcd $(M+H)^+$ = 677.2).

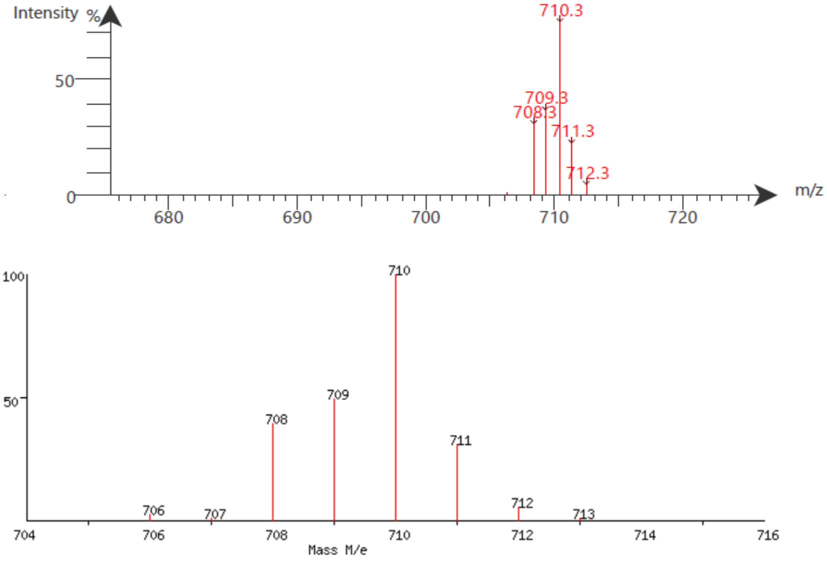

Figure S10. Experimental (top) and theoretical (bottom) mass spectrum of $[Pb(DOTA\text{-}3Py)]^+$ (*m/z* calcd $(M)^+$ = 710.3).

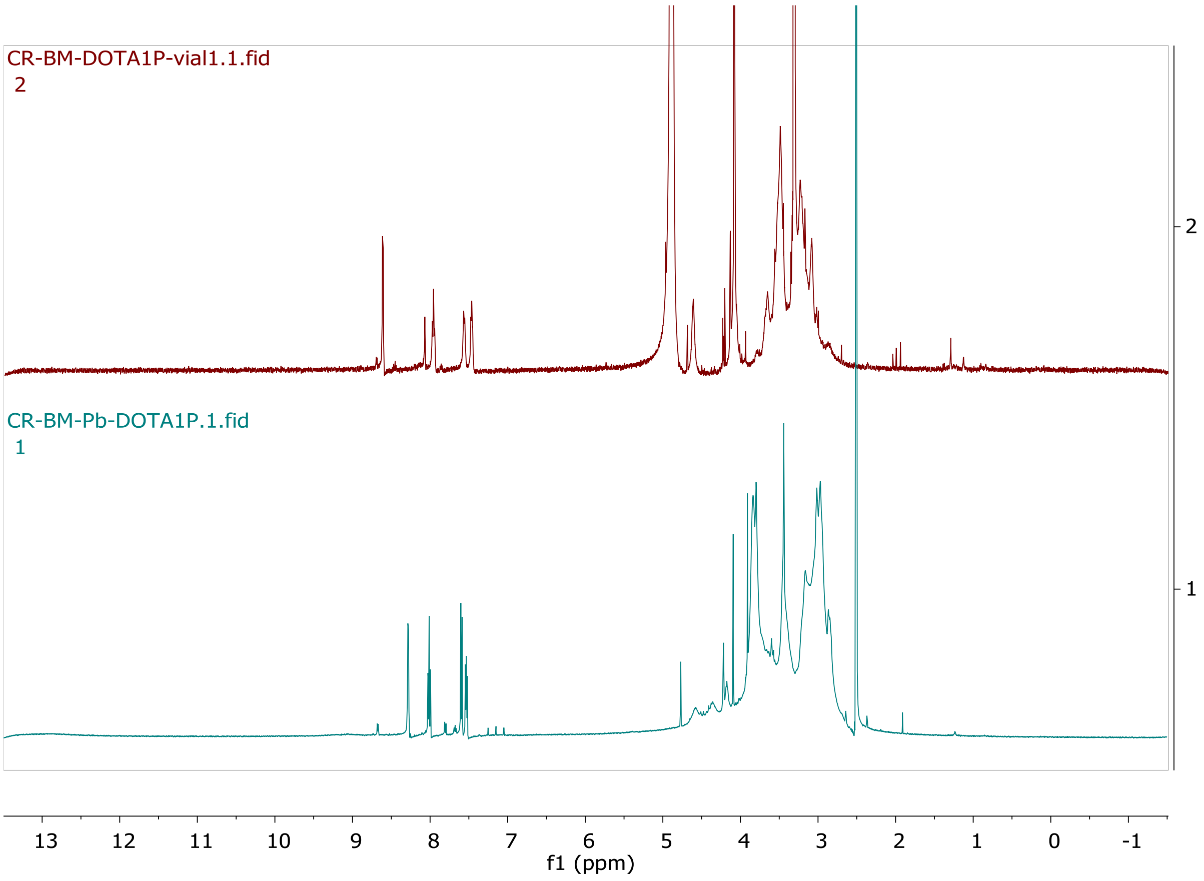

Figure S11. (top) ^1H NMR of DOTA-1Py (500 MHz, MeOD, 25°C). (bottom) ^1H NMR of $[Pb(DOTA\text{-}1Py)]^-$ (500 MHz, DMSO-d_6, 25°C).

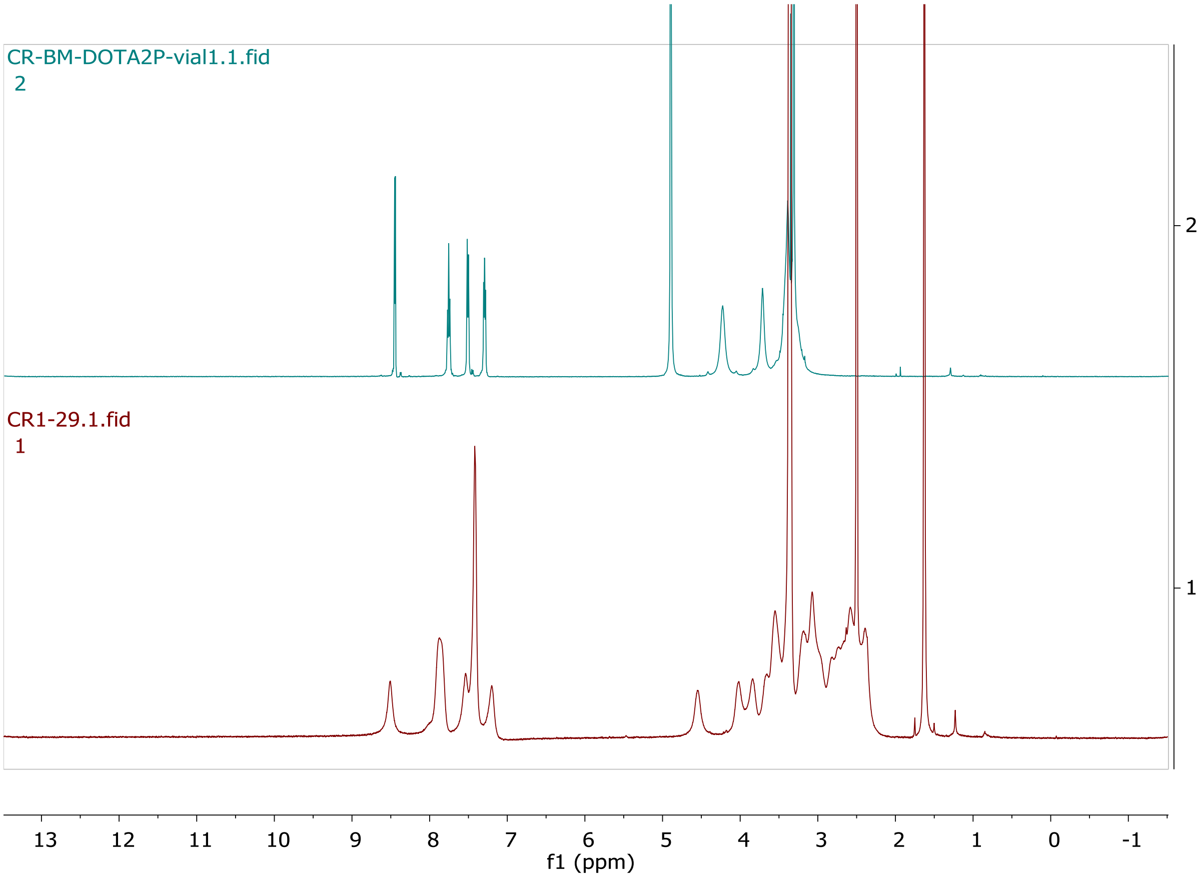

Figure S12. (top) ^{1}H NMR of DOTA-2Py (500 MHz, MeOD, 25°C). (bottom) ^{1}H NMR of [Pb(DOTA-2Py)] (500 MHz, DMSO-d_6, 25°C).

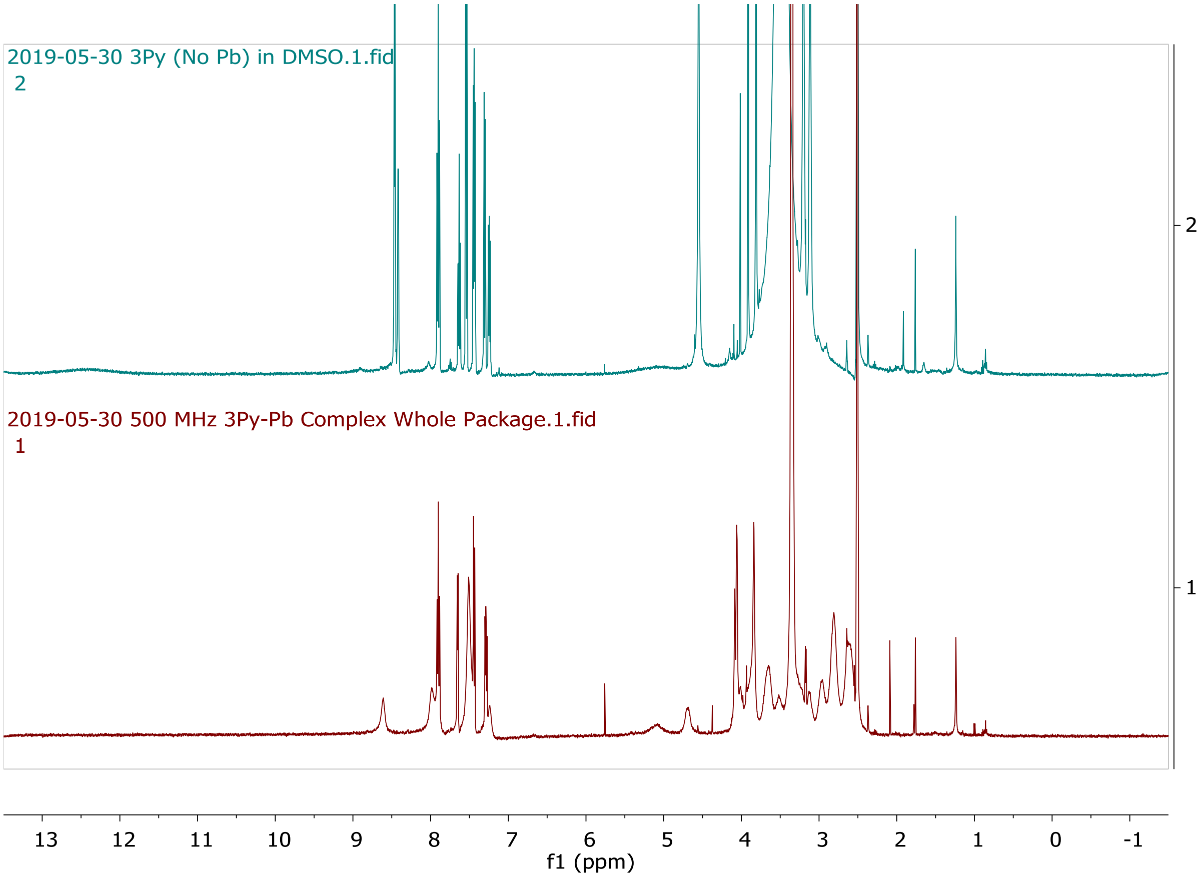

Figure S13. (top) ^{1}H NMR of DOTA-3Py (500 MHz, DMSO-d_6, 25°C). (bottom) ^{1}H NMR of $[Pb(DOTA\text{-}3Py)]^-$ (500 MHz, DMSO-d_6, 25°C).

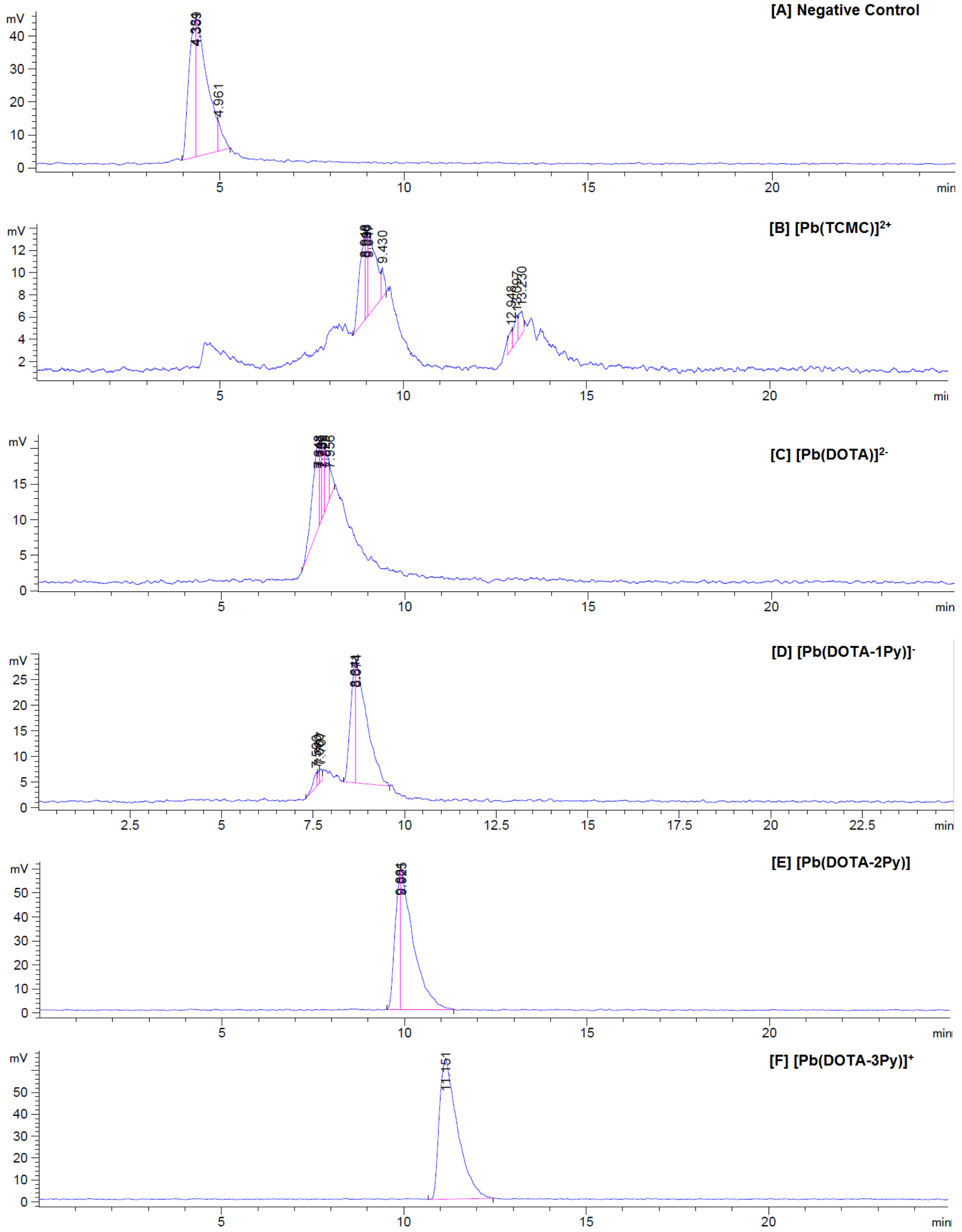

Figure S14. Radio-HPLC traces of ^{203}Pb-complex human serum stability studies after 72 h of incubation at 37°C. The retention time of free ^{203}Pb [A] was 4.4 minutes, labeled TCMC [B] was 9.0 minutes, labeled DOTA [C] was 9.6 minutes, labeled DOTA-1Py [D] was 8.6 minutes, labeled DOTA-2Py was 9.9 minutes, and labeled DOTA-3Py was 11.1 minutes.

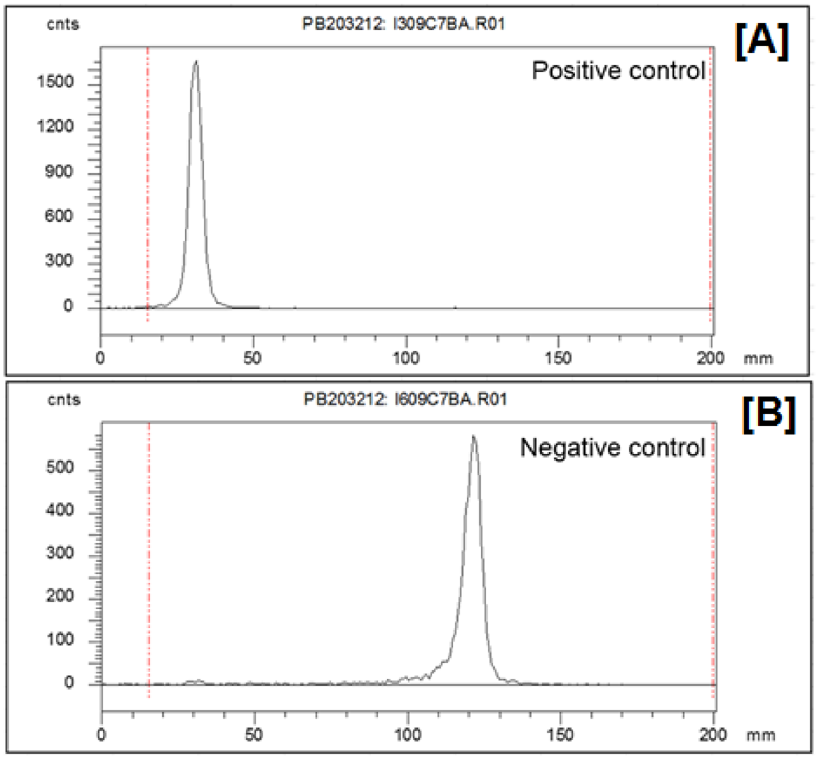

Figure S15. Representative positive and negative control iTLC radio-chromatograms for ^{203}Pb radiolabelling. [A] 10^{-4} M DOTA-3Py labelled with ~50 kBq ^{203}Pb, [B] unlabelled ^{203}Pb, at 1 h aliquot spotted onto SA iTLC plates, developed using EDTA (50 mM, pH 5.0) as the mobile phase.

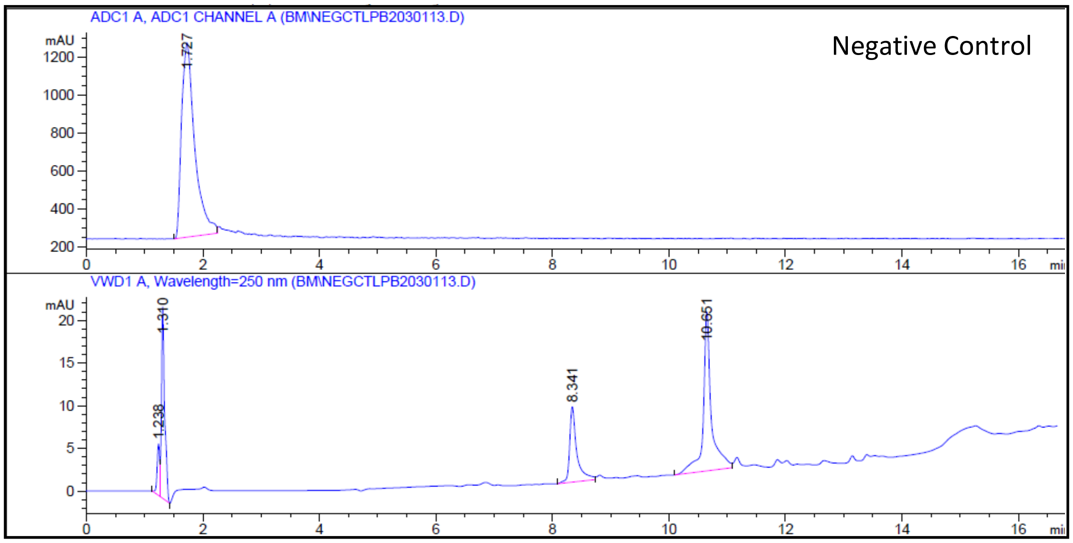
Negative Control
ADC1 A, ADC1 CHANNEL A (BM\NEGCTLPB2030113.D)
mAU
1.727
min
VWD1 A, Wavelength=250 nm (BM\NEGCTLPB2030113.D)
mAU
1.238
1.310
8.341
10.651
min

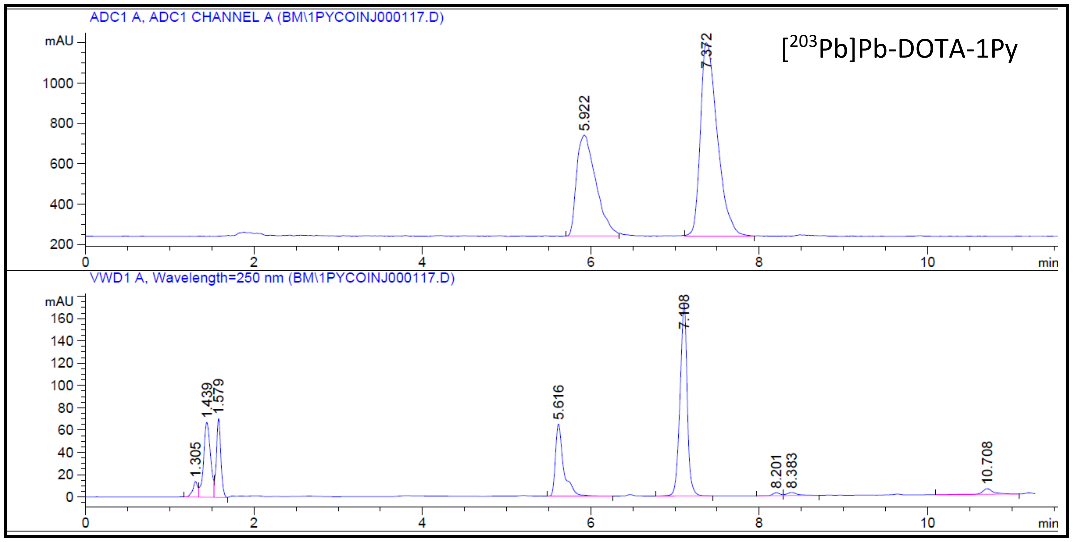
[203Pb]Pb-DOTA-1Py
ADC1 A, ADC1 CHANNEL A (BM\1PYCOINJ000117.D)
mAU
5.922
7.372
min
VWD1 A, Wavelength=250 nm (BM\1PYCOINJ000117.D)
mAU
1.305
1.439
1.579
5.616
7.108
8.201
8.383
10.708
min

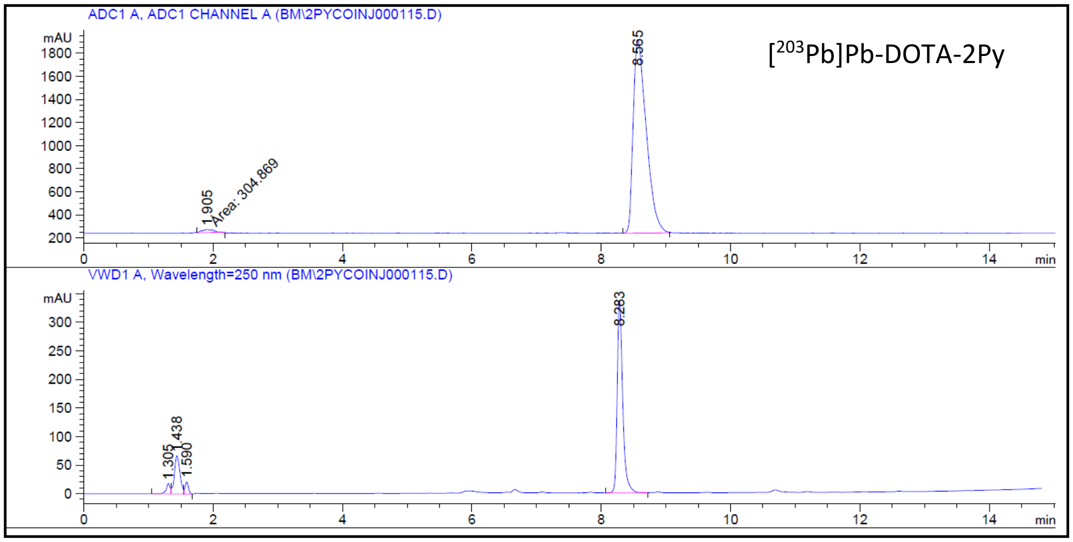
[203Pb]Pb-DOTA-2Py
ADC1 A, ADC1 CHANNEL A (BM\2PYCOINJ000115.D)
mAU
1.905
Area: 304.869
8.565
min
VWD1 A, Wavelength=250 nm (BM\2PYCOINJ000115.D)
mAU
1.305
1.438
1.590
8.283
min

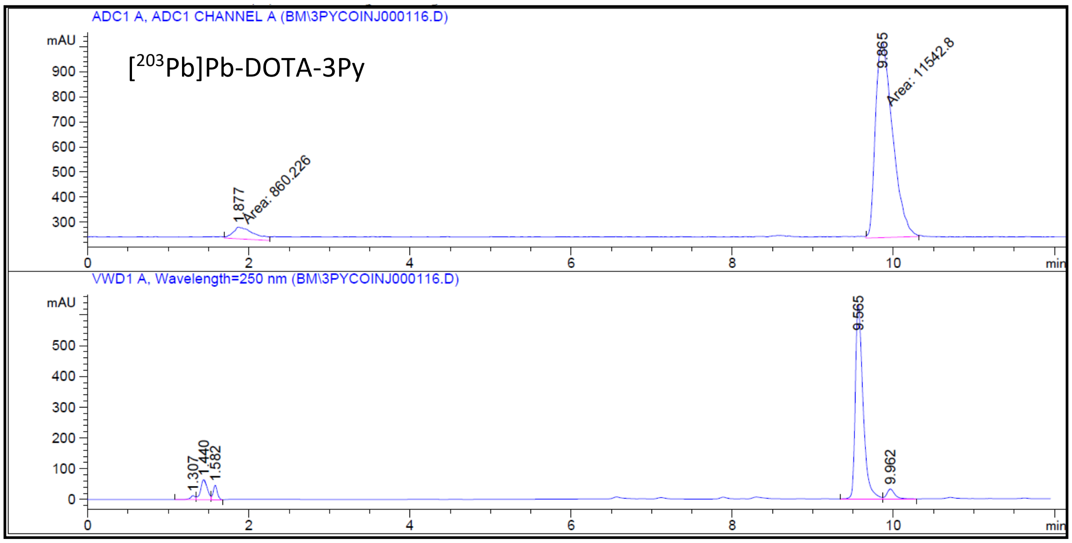

Figure S16. Radioactive (top) and UV (absorbance at 250 nm; bottom) RP-HPLC chromatograms of co-injected ^{203}Pb- and ^{nat}Pb-DOTA-xPy (x = 1 – 3) complexes. ^{203}Pb labeling of ligands at 10^{-4} M, NH_4OAc (1M, pH 7), room temperature, 1 hour. Retention time shift of ~0.3 min between radioactive and UV traces occur due to the detector and instrument set-up.
